# Beyond resorption-driven coupling: a multi-layered framework for osteoclast–osteoblast communication and its therapeutic consequences

**DOI:** 10.3389/fendo.2026.1821292

**Published:** 2026-04-20

**Authors:** Hongtao Qiu, Shiming Liu, Yang Jiang, Yuwen Lai, Qing Lin, Aisi Huang

**Affiliations:** Shenzhen Bao’an Chinese Medicine Hospital, Guangzhou University of Chinese Medicine, Shenzhen, China

**Keywords:** bone remodeling, cellular communication, coupling factors, osteoblast, osteoclast, skeletal homeostasis

## Abstract

Skeletal homeostasis depends on tightly coordinated communication between osteoclasts and osteoblasts, yet the molecular logic governing this coupling remains incompletely understood. This review reframes the osteoclast–osteoblast relationship by integrating developmental biology, molecular signaling, and translational perspectives into a unified analytical framework. We first trace the developmental origins of osteoclasts across embryonic hematopoietic waves, presenting evidence that ontogenetic heterogeneity—rather than being a developmental relic—actively shapes the coupling capacity of osteoclast populations throughout life. We then examine the hierarchical differentiation cascade of the osteoblast lineage, emphasizing how the adipo-osteo switch and hormonal regulation at each differentiation stage create multiple points of vulnerability and therapeutic opportunity. A central argument of this review is that pre-osteoclasts function as major, and potentially dominant, coupling effectors in bone remodeling. Operating through a secretome that includes sphingosine-1-phosphate, PDGF-BB, and afamin, these mononuclear precursors coordinate osteoblast recruitment and vascularization independently of bone resorption. However, the relative contribution of pre-osteoclast-derived signals versus other coupling mechanisms likely varies by skeletal site, age, and pathological context. We systematically dissect three core signaling cascades—BMP, sphingolipid/sclerostin, and WNT—and argue that their functional convergence creates a robust yet tunable communication network. We further evaluate recently identified coupling factors including cardiotrophin-1, SLIT3, C3a, and CTHRC1, alongside surface-mediated and vesicle-based communication systems. Finally, we critically assess current therapeutic strategies through the lens of coupling biology, proposing that the persistent failure to develop truly disease-modifying skeletal therapies stems from an incomplete appreciation of the multi-layered nature of osteoclast–osteoblast communication. Collectively, this review establishes that the anabolic and resorptive functions of the osteoclast lineage are mechanistically separable and proposes that therapeutic strategies aimed at expanding the coupling-competent pre-osteoclast pool—rather than broadly suppressing or stimulating remodeling—represent a paradigm shift toward next-generation skeletal therapies that preserve, rather than disrupt, the endogenous coupling network.

## Graphical Abstract

**Figure:**
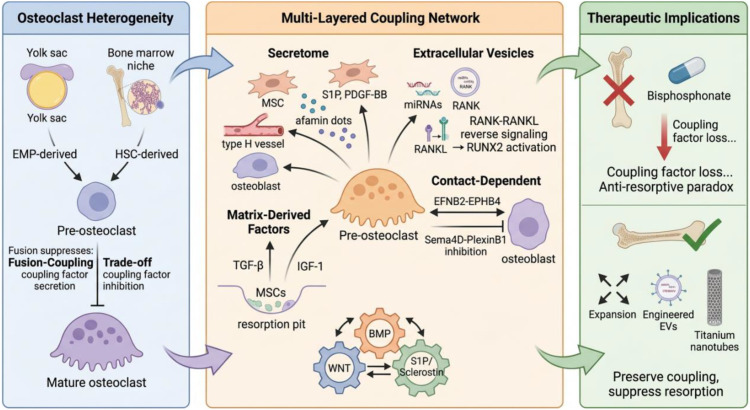

## 1 Introduction

Bone is often described as a dynamic organ, yet this characterization understates the remarkable precision with which its cellular inhabitants coordinate their activities. At any given moment, approximately two million remodeling sites are active across the adult skeleton, each requiring exact temporal and spatial coordination between bone-resorbing osteoclasts and bone-forming osteoblasts (1, 2). When this coordination fails—as occurs in osteoporosis, a condition affecting hundreds of millions worldwide—the consequences extend far beyond skeletal fragility to encompass diminished mobility, chronic pain, and substantially increased mortality (3, 4).The conceptual framework for understanding osteoclast–osteoblast communication has evolved considerably over the past two decades. Early models envisioned a relatively simple sequential process: osteoclasts resorb bone, release matrix-embedded growth factors, and osteoblasts passively respond by filling the resorption cavity. This “resorption-driven coupling” hypothesis, while not incorrect, captured only one dimension of a far more elaborate communication system.

We now recognize that osteoclasts and osteoblasts engage in a continuous, multi-modal dialogue involving direct cell–cell contact, secreted coupling factors, extracellular vesicles, and matrix-derived signals (5, 6). More fundamentally, the discovery that pre-osteoclasts—mononuclear precursors that have not yet acquired resorptive capacity—can act as potent stimulators of bone formation under specific experimental conditions has challenged the assumption that coupling necessarily requires prior resorption (7, 8). In parallel, advances in lineage-tracing technology have revealed that the osteoclast compartment is far more heterogeneous than previously appreciated. The persistence of embryonically derived osteoclasts into adulthood, alongside continuously replenished hematopoietic stem cell (HSC)–derived populations, raises a provocative question: does developmental origin influence the coupling capacity of an osteoclast? If so, the implications for both basic biology and therapeutic strategy would be profound.

This review addresses these questions by synthesizing current knowledge across three interconnected domains. We begin with the developmental biology of osteoclasts and osteoblasts, establishing how cellular heterogeneity is generated. We then dissect the molecular communication mechanisms between these lineages, from core signaling cascades to recently identified coupling factors. Finally, we evaluate therapeutic strategies through the lens of coupling biology, arguing that the limitations of current treatments are predictable consequences of disrupting the very communication networks that maintain skeletal homeostasis. Importantly, we move beyond conceptual reframing to propose an operationally defined analytical framework that differs from the prevailing “resorption-driven coupling” model in three specific respects: it incorporates developmental origin as a variable influencing coupling output, it defines the pre-osteoclast as a functionally distinct coupling state governed by a fusion–coupling trade-off, and it organizes coupling mechanisms into a hierarchically layered network whose architecture generates specific, experimentally testable predictions—which we articulate at the relevant points throughout this review.

## 2 Developmental origins of osteoclasts: from embryonic waves to lifelong heterogeneity

The question of where osteoclasts come from might seem straightforward—they are derived from hematopoietic precursors and differentiated under the influence of M-CSF and RANKL. However, this conventional answer obscures a more complex reality. Mammalian osteoclastogenesis is rooted in multiple waves of embryonic hematopoiesis, each generating precursor populations with distinct molecular identities, migratory behaviors, and—potentially—coupling functions. It should be noted at the outset that the evidence presented in this section derives predominantly from murine lineage-tracing studies, and the extent to which these findings can be generalized to human osteoclast biology remains an open question that we address explicitly at the end of this section.

### 2.1 Layered hematopoiesis and osteoclast ontogeny

Mammalian hematopoiesis does not arise as a single developmental event but proceeds through temporally and spatially distinct waves (9, 10). The earliest—termed the primitive wave—emerges in yolk sac blood islands, generating nucleated erythroid cells, megakaryocytes, and primitive macrophages (11, 12). Shortly thereafter, erythromyeloid progenitors (EMPs) arise from the yolk sac hemogenic endothelium at approximately embryonic day (E) 7–7.5 (13, 14). These EMPs differentiate into CSF1R-expressing macrophages through MYB-independent pathways by E8.5, bypassing classical monocytic intermediates entirely (11, 15). Following the establishment of vascular connections between the yolk sac and embryonic circulation around E8.25 (16), these primitive macrophages colonize developing tissues critical for skeletal morphogenesis, including the craniofacial complex and long bones (15, 17).

The significance of this early colonization cannot be overstated. Through cell fusion, EMP-derived macrophages generate the first functional osteoclasts, which are indispensable for dental eruption, craniofacial shaping, and long bone modeling (18). These embryonic osteoclasts are therefore not passive bystanders but active architects of skeletal patterning.

A second wave of hematopoiesis emerges around E8.25, characterized by MYB-dependent “late” EMPs that migrate from the yolk sac to the fetal liver (19–21). Within this hepatic niche, they give rise to monocytes that further contribute to osteoclast pools. In parallel, definitive HSC precursors emerge in the aorta–gonad–mesonephros region at approximately E10.5, subsequently expanding in the fetal liver before colonizing the bone marrow to establish lifelong hematopoiesis (22, 23). HSC-derived cells progressively contribute to osteoclast formation during late embryogenesis, postnatal growth, and adulthood (24).

### 2.2 Persistence rather than replacement

Perhaps the most conceptually important finding in recent osteoclast biology is that embryonically derived populations are not fully replaced after birth. Lineage-tracing studies using yolk sac–specific markers have demonstrated that EMP-derived macrophages persist within adult tissues, including the spleen and bone marrow, functioning as a long-term reservoir of osteoclast precursors (15, 25). During fracture healing, these embryonically derived cells are actively recruited to injury sites where they contribute substantially to osteoclast formation (18).

This persistence creates a situation in which osteoclasts of fundamentally different developmental origins coexist at active remodeling sites. Because osteoclast formation requires cell–cell fusion, multinucleated osteoclasts can harbor nuclei of both EMP and HSC origin (18).

We propose that this mixed-origin architecture may have functional consequences for coupling. If embryonic-derived and HSC-derived precursors express different complements of coupling factors—a hypothesis that remains to be formally tested—then the ratio of these populations at a given remodeling site could influence the quality and quantity of anabolic signals received by neighboring osteoblasts. This framework would provide a developmental explanation for the site-specific and age-dependent variations in bone remodeling efficiency observed clinically. This hypothesis generates a concrete testable prediction: if EMP-derived and HSC-derived osteoclast precursors are isolated from the same skeletal site using lineage-specific reporters and subjected to single-cell secretome profiling, their coupling factor repertoires (S1P, PDGF-BB, CT-1, WNT10B, sclerostin) should differ systematically. A corollary prediction is that the ratio of EMP-derived to HSC-derived osteoclasts at a given site shifts with aging in a manner that correlates with local bone formation rate. Falsification of either prediction would necessitate revision of the ontogeny-coupling dimension of our framework.

An important caveat is that the evidence supporting this framework derives almost entirely from murine models. The lineage-tracing tools that have been instrumental in demonstrating EMP-derived osteoclast persistence—including yolk sac-specific Cre drivers and fate-mapping reporters—are unavailable for human studies, and direct evidence for the long-term persistence of embryonically derived osteoclast populations in adult human bone does not currently exist.

Several fundamental differences between murine and human biology further complicate direct extrapolation. Human skeletal development occurs over a substantially longer timeframe, with distinct patterns of hematopoietic ontogeny: while the general architecture of layered hematopoiesis is conserved across mammals, the precise timing, anatomical locations, and molecular regulators of each wave differ between species. For example, the relative contributions of yolk sac versus aorta-gonad-mesonephros-derived hematopoiesis to postnatal tissue-resident macrophage populations may not be identical in mice and humans. Additionally, the remodeling biology of cortical bone—which constitutes a larger proportion of the human skeleton relative to the mouse—has been less thoroughly characterized in the context of osteoclast ontogeny.

Indirect evidence from human studies is consistent with, but does not confirm, the persistence model. Observations from sex-mismatched bone marrow transplantation have demonstrated that tissue-resident macrophage populations in various organs are not fully replaced by donor-derived cells even years after transplantation, suggesting that embryonically derived myeloid populations can persist in humans as they do in mice. However, whether this principle extends specifically to bone-resident osteoclast precursors, and whether any such persisting populations retain distinct coupling properties, remains entirely unknown. The ontogeny-coupling hypothesis proposed here should therefore be understood as a conceptual framework generated primarily from murine data that requires rigorous validation in human systems before its clinical implications can be assessed.

### 2.3 Convergent molecular programs in osteoclastogenesis

Regardless of their developmental origin, osteoclast precursors ultimately engage a conserved differentiation program centered on M-CSF and RANKL signaling. M-CSF, acting through CSF1R, ensures precursor proliferation and survival (24), while RANKL activates downstream transcriptional programs via NF-κB and NFATc1 (26). However, emerging evidence from murine systems suggests that precursor origin influences sensitivity to these signals, shaping differentiation kinetics and osteoclastogenic efficiency. Whether analogous origin-dependent differences exist among human osteoclast precursors has not been investigated.

The plasticity of the myeloid lineage adds further complexity. Multiple myeloid populations—including CSF1R^+^ bone marrow progenitors and dendritic cell subsets—retain osteoclastogenic potential under permissive conditions. This indicates that osteoclast differentiation represents a context-dependent fate decision rather than a rigid lineage endpoint.

The final step of osteoclastogenesis—cell fusion—is regulated by DC-STAMP, CD47, and vacuolar ATPase components, which coordinate membrane recognition, cytoskeletal rearrangement, and multinuclear stability. Critically, fusion is a selective process that integrates developmental and environmental information, making it a potential control point for coupling factor expression. Inflammatory cues profoundly modulate each stage of this process: pro-inflammatory cytokines amplify RANKL signaling, accelerate fusion, and can skew osteoclast populations toward heightened resorptive activity at the expense of coupling function. This represents a critical interface between immune activation and skeletal homeostasis that is particularly relevant to inflammatory bone diseases.

## 3 The osteoblast lineage: a hierarchical cascade with multiple regulatory checkpoints

If osteoclast biology defines the “demand” side of remodeling, the osteoblast lineage determines the “supply.” The generation of functional osteoblasts follows a multi-step hierarchical cascade that is tightly regulated at each transition point (27). Understanding this cascade is essential for appreciating how osteoclast-derived coupling signals act on specific stages of osteoblast maturation—and where the process is most vulnerable to pathological disruption.

### 3.1 MSC lineage allocation: the adipo-osteo decision

Mesenchymal stem cells (MSCs) serve as the fundamental reservoir for skeletal regeneration, residing in bone marrow, periosteum, and endosteum (28). Characterized by specific surface markers (CD73^+^, CD90^+^, CD105^+^, lineage negative) (29), their most consequential biological property is their capacity for competitive lineage allocation.

The “adipo-osteo switch” represents a critical determinant of skeletal integrity (30). Activation of Runx2 and Wnt/β-catenin signaling drives osteogenesis while suppressing the adipogenic regulator PPARγ; conversely, PPARγ activation promotes adipocyte differentiation at the expense of the osteoblast pool. In aging and osteoporosis, this balance shifts decisively toward adipogenesis. The resulting accumulation of marrow adipose tissue is not merely a passive replacement of bone-forming cells but actively impairs hematopoiesis and regeneration through lipotoxic effects and altered paracrine signaling (31). This observation carries an important therapeutic implication: effective treatment of age-related bone loss must address this upstream lineage bias, not merely stimulate existing osteoblasts (29).

### 3.2 Osteoprogenitor expansion and niche-specific behavior

Between the quiescent MSC pool and mature osteoblasts lies a heterogeneous population of osteoprogenitor cells (OPCs) that function as a transit-amplifying compartment (32). Their behavior is highly context-dependent, varying by anatomical location and the nature of osteogenic demand.

The characterization of OPC populations has advanced considerably through lineage-tracing and single-cell transcriptomic approaches, revealing a diverse array of progenitor subsets defined by distinct molecular markers. These include Nestin-expressing perivascular progenitors closely associated with the hematopoietic niche (33), Leptin receptor (LepR)-positive stromal cells that represent a major source of osteoblasts and adipocytes in adult bone marrow (34), Gremlin 1 (Grem1)-expressing progenitors that function as self-renewing osteochondroreticular stem cells (35), Gli1-positive cells enriched in the metaphyseal and periosteal regions (36), Mx1-expressing cells that contribute to osteoblast formation during homeostasis and injury (37), Osterix/Sp7-lineage progenitors at various stages of commitment (38), Cathepsin K-expressing periosteal stem cells recently identified as contributors to intramembranous bone formation (39), and Prrx1-positive limb mesenchymal progenitors (40). This diversity underscores that the OPC compartment is not a uniform transit-amplifying population but rather comprises functionally specialized subsets with distinct anatomical distributions, self-renewal capacities, and differentiation potentials.

Among these populations, α-SMA-expressing periosteal progenitors have been particularly well characterized for their role in fracture repair (41) and their pronounced mechanosensitivity: mechanical loading specifically triggers their proliferation and differentiation (42). Hormonal regulation is equally important across OPC subsets. Estrogen receptor alpha (ERα) signaling maintains OPC proliferation and viability; its loss depletes the cortical bone progenitor pool and uncouples resorption from compensatory formation (43, 44). The OPC compartment therefore represents a point where hormonal deficiency, mechanical disuse, and coupling factor deficiency can converge to produce the bone loss characteristic of osteoporosis. Importantly, different OPC subsets may exhibit differential responsiveness to osteoclast-derived coupling signals—a possibility that has not been systematically explored but could have significant implications for understanding site-specific variations in remodeling efficiency.

### 3.3 Terminal differentiation and fate decisions

The transition from OPC to mature osteoblast marks a shift from proliferation to massive biosynthetic activity, with production of type I collagen osteoid and its mineralization requiring substantial metabolic energy and enzymatic control via alkaline phosphatase (45, 46).

Upon completing their synthetic function, osteoblasts face a tripartite fate decision: apoptosis, transformation into quiescent bone-lining cells, or incorporation into the mineralized matrix as osteocytes (47). The osteocyte transition is particularly consequential, as it creates the mechanosensory network that regulates subsequent remodeling cycles. The proportion of osteoblasts successfully transitioning to osteocytes determines the long-term mechanosensitivity of the bone—and, as we discuss next, this entire cascade is subject to regulation by signals originating from the osteoclast lineage.

## 4 Pre-osteoclasts as coupling effectors: decoupling anabolism from resorption

The conventional model of bone coupling positions resorption as the initiating event: osteoclasts degrade bone matrix, release embedded growth factors, and osteoblasts respond. While this sequence is well supported, it cannot fully explain several experimental observations. Most notably, conditions that arrest osteoclast differentiation at the mononuclear stage—preventing resorption entirely—can paradoxically increase bone formation (48). This section examines the evidence that pre-osteoclasts function as major coupling effectors, operating through a specialized secretome that is mechanistically distinct from resorption-derived coupling. We note that while the experimental evidence for resorption-independent coupling is compelling, the relative physiological contribution of pre-osteoclast-derived signals versus resorption-dependent mechanisms in normal adult remodeling remains an active area of investigation.

### 4.1 The fusion–coupling trade-off

Pre-osteoclasts are a primary source of sphingosine-1-phosphate (S1P), which acts as a chemoattractant and survival factor for MSCs (49, 50) (the receptor-level complexity of S1P signaling is detailed in Section 6.5). The BMP/SMAD signaling cascade provides a compelling mechanistic illustration of the fusion–coupling trade-off. Deletion of SMAD1/5 in the osteoclast lineage arrests differentiation at the fusion stage, leading to accumulation of fusion-incompetent pre-osteoclasts that secrete elevated levels of S1P and drive robust osteogenic responses (51, 52). This reveals a biological trade-off that we consider conceptually important: the molecular machinery driving fusion may actively antagonize secretory coupling function. If correct, this would position the pre-osteoclast not merely as a transitional intermediate but also as a distinct functional state with important biological roles—a concept with significant therapeutic implications, as strategies that expand the pre-osteoclast pool without promoting fusion could theoretically enhance bone formation without increasing resorption. It should be noted, however, that this trade-off has been primarily demonstrated in genetic models with SMAD1/5 deletion, and its generalizability to physiological remodeling contexts requires further validation. Nevertheless, this trade-off yields a generalizable and falsifiable prediction: any intervention that arrests osteoclast differentiation at the mononuclear stage—whether through DC-STAMP inhibition, CD47 blockade, or other disruption of fusion machinery—should enhance net coupling output, provided the pre-osteoclast pool is maintained. This prediction would be refuted by fusion-blocking interventions that simultaneously reduce pre-osteoclast viability or suppress their secretory function, an outcome that would indicate the trade-off is context-dependent rather than a general principle.

### 4.2 Orchestrating the osteo-vascular niche

Pre-osteoclasts coordinate both cellular recruitment and vascular support for bone formation, establishing what can be termed an “osteo-vascular niche.” Platelet-derived growth factor-BB (PDGF-BB) is a pivotal mediator: pre-osteoclast-derived PDGF-BB induces formation of type H vessels, a specialized capillary subtype spatially and temporally coupled to osteogenesis (53–56). The tripartite interaction—pre-osteoclasts, endothelial cells, and osteoblasts—creates a feed-forward loop in which increased pre-osteoclast density drives vascular expansion and, consequently, bone mass accrual (57, 58).

The clinical relevance of this pathway is illustrated by bisphosphonate biology. Zoledronate has been shown to suppress angiogenesis and osteogenesis through multiple mechanisms, including direct anti-angiogenic effects on endothelial cells. Among these mechanisms, the reduction of pre-osteoclast numbers and their PDGF-BB secretion has been identified as one significant contributing pathway (59). While bisphosphonates exert pleiotropic effects on the bone microenvironment, the loss of pre-osteoclast-derived coupling signals likely contributes to why long-term anti-resorptive therapy, despite preventing bone loss, fails to restore bone quality. The concurrent elimination of multiple coupling factor sources—including but not limited to the pre-osteoclast pool—provides a partial molecular explanation for the progressive decline in bone formation observed during prolonged treatment.

**Figure 1:**
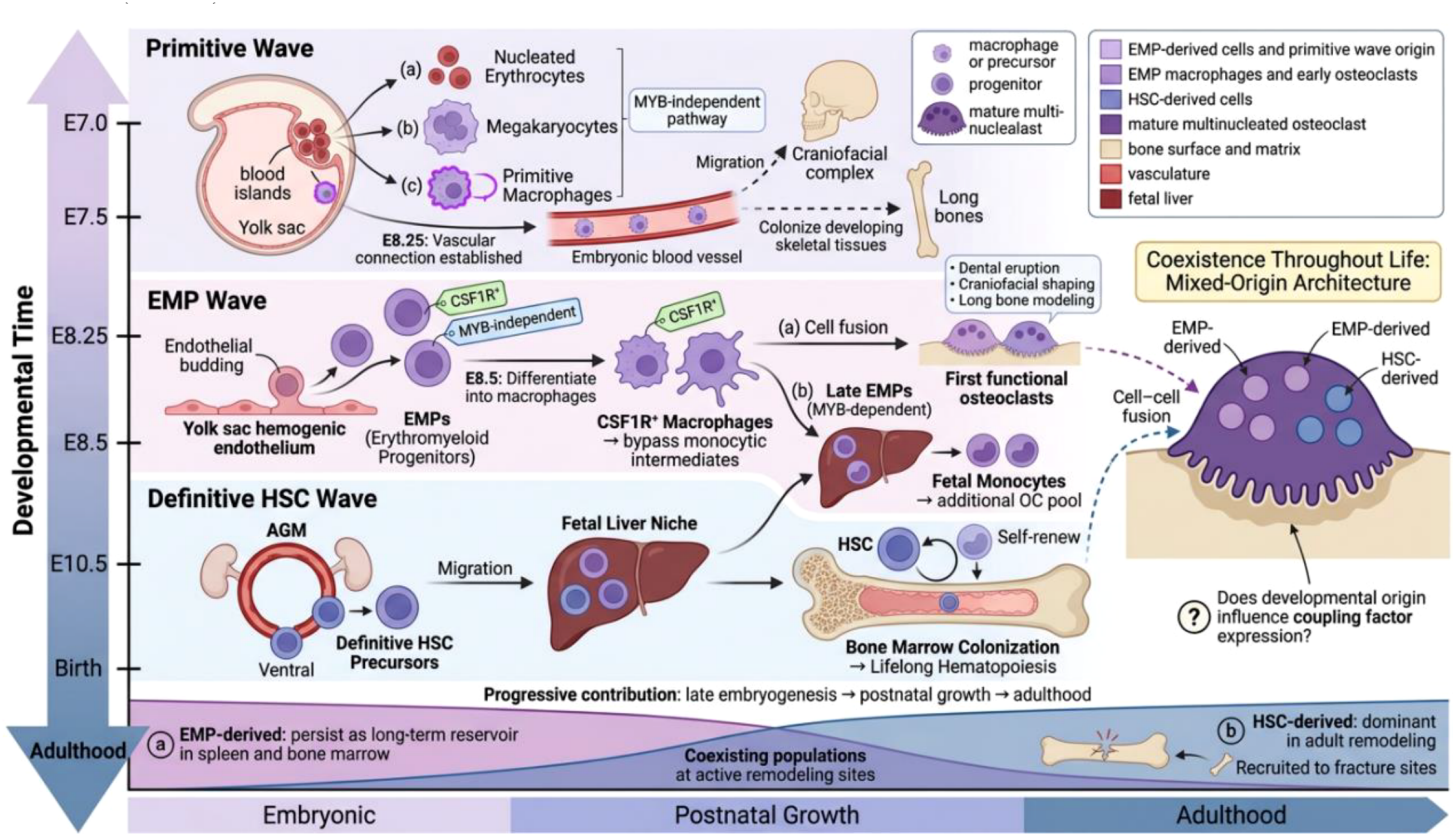
Developmental origins of osteoclasts: from embryonic waves to lifelong coexistence. Three temporally distinct hematopoietic waves—primitive (E7.0), EMP (E7.5–E8.5), and definitive HSC (E10.5)—contribute to osteoclast formation. EMP-derived macrophages generate the first functional osteoclasts essential for skeletal patterning. Critically, EMP-derived populations persist into adulthood rather than being fully replaced, resulting in multinucleated osteoclasts of mixed developmental origin at active remodeling sites. The bottom panel illustrates the relative contributions of EMP-derived (purple) and HSC-derived (blue) populations across the lifespan. See Sections 2.1–2.2 for details.

Targeting Siglec-15 offers a mechanistically distinct alternative. Siglec-15 is a DAP12-associated transmembrane lectin selectively expressed on osteoclast lineage cells, where it functions as a positive regulator of osteoclast multinucleation and maturation by modulating RANKL-induced phosphatidylinositol 3-kinase/Akt and Erk signaling pathways (60, 61). Unlike bisphosphonates and denosumab, which reduce osteoclast lineage cells broadly, antibody-mediated Siglec-15 inhibition specifically blocks the transition from mononuclear pre-osteoclasts to mature multinucleated osteoclasts. This results in an expanded pool of TRAP^+^ mononuclear cells that retain their secretory coupling function, including robust PDGF-BB production, while mature osteoclast formation and resorptive activity are suppressed. In murine models, anti-Siglec-15 antibody treatment simultaneously promoted bone formation and suppressed resorption, and enhanced fracture healing by increasing type H vessel density at repair sites (60, 61). This pharmacological profile—anti-resorptive without being anti-anabolic—provides direct *in vivo* evidence for the fusion–coupling trade-off described above and positions Siglec-15 as a promising therapeutic target that exploits, rather than disrupts, the coupling network. Pre-osteoclast-derived afamin provides additional chemotactic specificity, mobilizing pre-osteoblasts via Akt signaling to ensure timely arrival of matrix-producing cells (62) (**Figure 1**).

## 5 Core signaling cascades in osteoclast–osteoblast communication

The coupling factors described above operate through three interconnected signaling networks that form the regulatory backbone of osteoclast–osteoblast communication. Rather than functioning as independent pathways, these cascades exhibit extensive cross-regulation, creating a signaling architecture that is both robust and tunable.

### 5.1 BMP signaling: integration and cross-regulation

The BMP pathway functions as more than a simple inducer of osteoblastogenesis—it operates as a molecular hub that integrates multiple signaling inputs. The receptor system uses a high-fidelity recognition mechanism: BMPR-IA targets BMP-2/4 while BMPR-IB selects BMP-4/7, enabling precise spatial control (63–65).

Beyond canonical Smad1/5/8 signaling that drives osteogenic transcription (66, 67), the pathway integrates non-canonical MAPK signals (p38 and JNK) to modulate ALP and osteocalcin expression (68, 69). Importantly, BMP-2 cross-regulates Wnt signaling by facilitating Dvl-1/Smad1 interaction, stabilizing β-catenin and linking BMP-driven differentiation to Wnt-driven proliferation (70). This convergence is reinforced by p38-mediated DLX5 activation that drives Osterix expression independently of Runx2 (71, 72).

**Figure 2:**
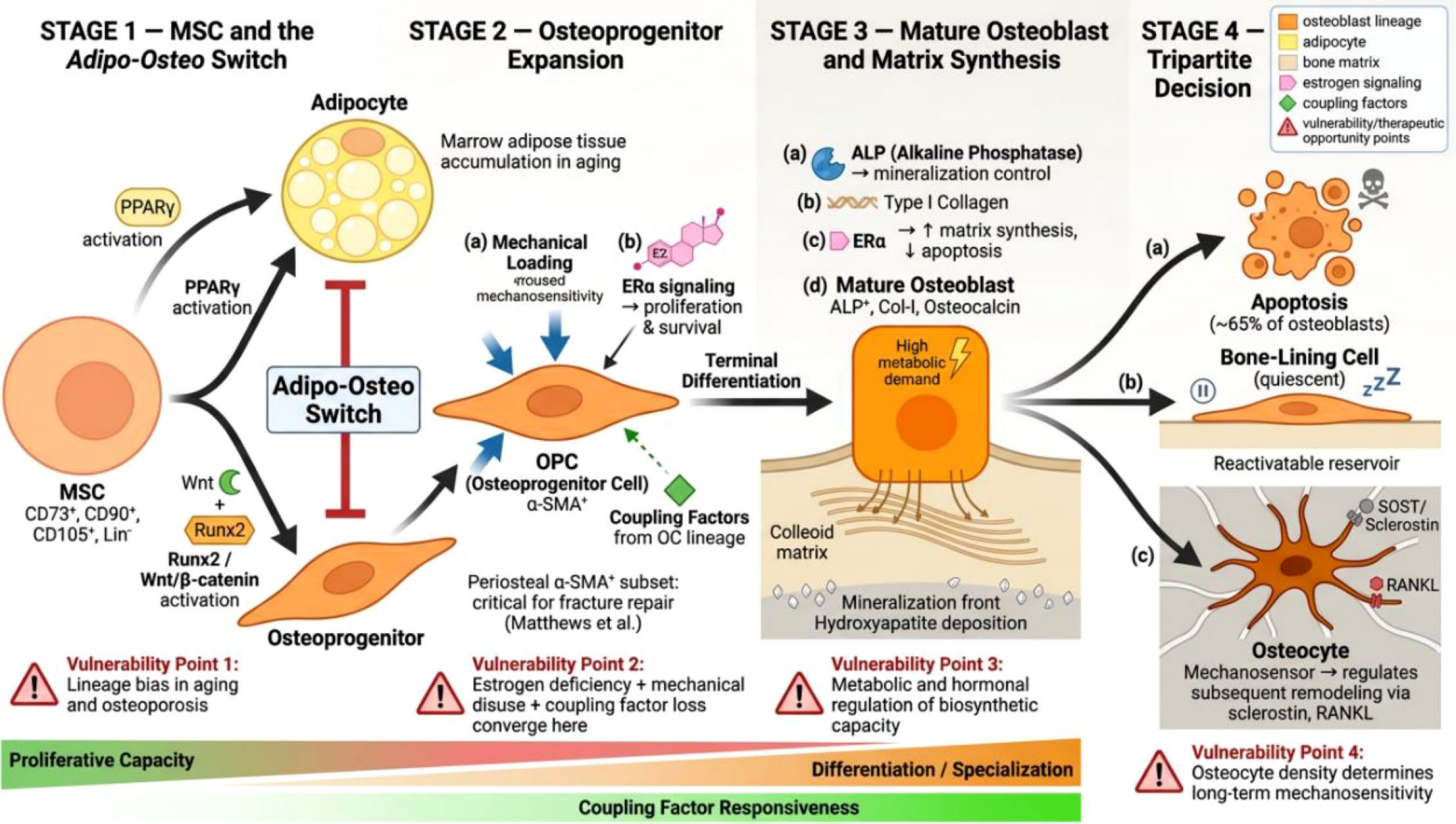
The osteoblast lineage: a hierarchical differentiation cascade with multiple vulnerability points. Osteoblast development proceeds from MSCs through osteoprogenitor cells (OPCs) to mature osteoblasts, which ultimately undergo apoptosis, become bone-lining cells, or differentiate into osteocytes. The adipo-osteo switch (Stage 1), mechanical loading and ERα signaling (Stage 2), metabolic demands of matrix synthesis (Stage 3), and osteocyte density (Stage 4) represent sequential vulnerability points (red triangles) where pathological disruption can produce bone loss. See Section 3 for details.

The pathway’s sensitivity is controlled by intrinsic negative feedback through Smurf ubiquitin ligases, which target key signaling intermediates for degradation and thereby function as a rheostat that prevents excessive BMP activation (73–75). Disruption of this feedback—through either Smurf1 or Smurf2 deficiency—leads to distinct skeletal pathologies, underscoring the importance of calibrated BMP signal strength for maintaining the RANKL/OPG balance and normal remodeling (73–75) (**Figure 2**).

### 5.2 The sphingolipid–sclerostin axis: built-in brakes

The sphingolipid and sclerostin axes exemplify a ‘push-pull’ dynamic within individual osteoclasts. S1P secretion (described mechanistically in Section 6.5) provides the anabolic drive, while sclerostin—a potent Wnt antagonist acting via LRP5/6 (76, 77)—provides the inhibitory counterbalance.

While osteocytes are unequivocally the principal source of sclerostin in bone, recent evidence has demonstrated that osteoclasts also produce sclerostin, albeit at substantially lower levels. This osteoclast-derived sclerostin may function as a local modulatory signal within the remodeling microenvironment, complementing the broader paracrine effects of osteocyte-derived sclerostin.

The age-dependence of osteoclast-derived sclerostin secretion is particularly instructive. Osteoclasts from older subjects exhibit heightened sclerostin release and diminished osteogenic support (50), suggesting that age-related changes in osteoclast secretory profiles may contribute, alongside increased osteocyte-derived sclerostin, to the progressive uncoupling of remodeling observed with aging. We suggest that at the local level, this dual-secretion model—S1P promoting and sclerostin inhibiting bone formation—may function as an additional feedback mechanism that helps prevent excessive bone deposition. Its age-related dysregulation, favoring the inhibitory arm, may represent one contributing mechanism—among others, including altered osteocyte mechanosensitivity and hormonal changes—underlying senile osteoporosis. This model predicts that the S1P-to-sclerostin ratio in osteoclast-conditioned media will shift progressively toward sclerostin dominance with increasing donor age, and that this shift will correlate with reduced osteoblast mineralization capacity in co-culture assays—a prediction amenable to direct experimental testing using age-stratified donor cohorts.

### 5.3 WNT networks: context determines function

WNT signaling operates through canonical (β-catenin-dependent) and non-canonical (Ca²^+^/JNK) pathways (78). The canonical pathway, in which ligands such as WNT3a and WNT10b stabilize β-catenin by inhibiting GSK-3β, drives osteoblast differentiation via Tcf/Lef transcription factors (79, 80).

WNT5A illustrates how a single ligand can promote differentiation and function across multiple skeletal cell types. Osteoblast-derived WNT5A acts on osteoclast precursors by binding ROR2, activating a JNK-c-Jun-Sp1 cascade that amplifies RANK expression and thereby promotes osteoclast differentiation (81). Similarly, osteoclast-derived WNT5A acts on osteoblasts through a specific serine phosphorylation that enables non-canonical ROR2 signaling, promoting osteoblast differentiation (82, 83). Rather than representing a bidirectional regulatory switch, these observations are consistent with a more straightforward principle: WNT5A promotes differentiation and functional maturation in whichever target cell it engages, with the downstream outcome—enhanced resorption or enhanced formation—determined by the identity of the receiving cell rather than by an intrinsic coupling logic within the ligand itself.

**Figure 3:**
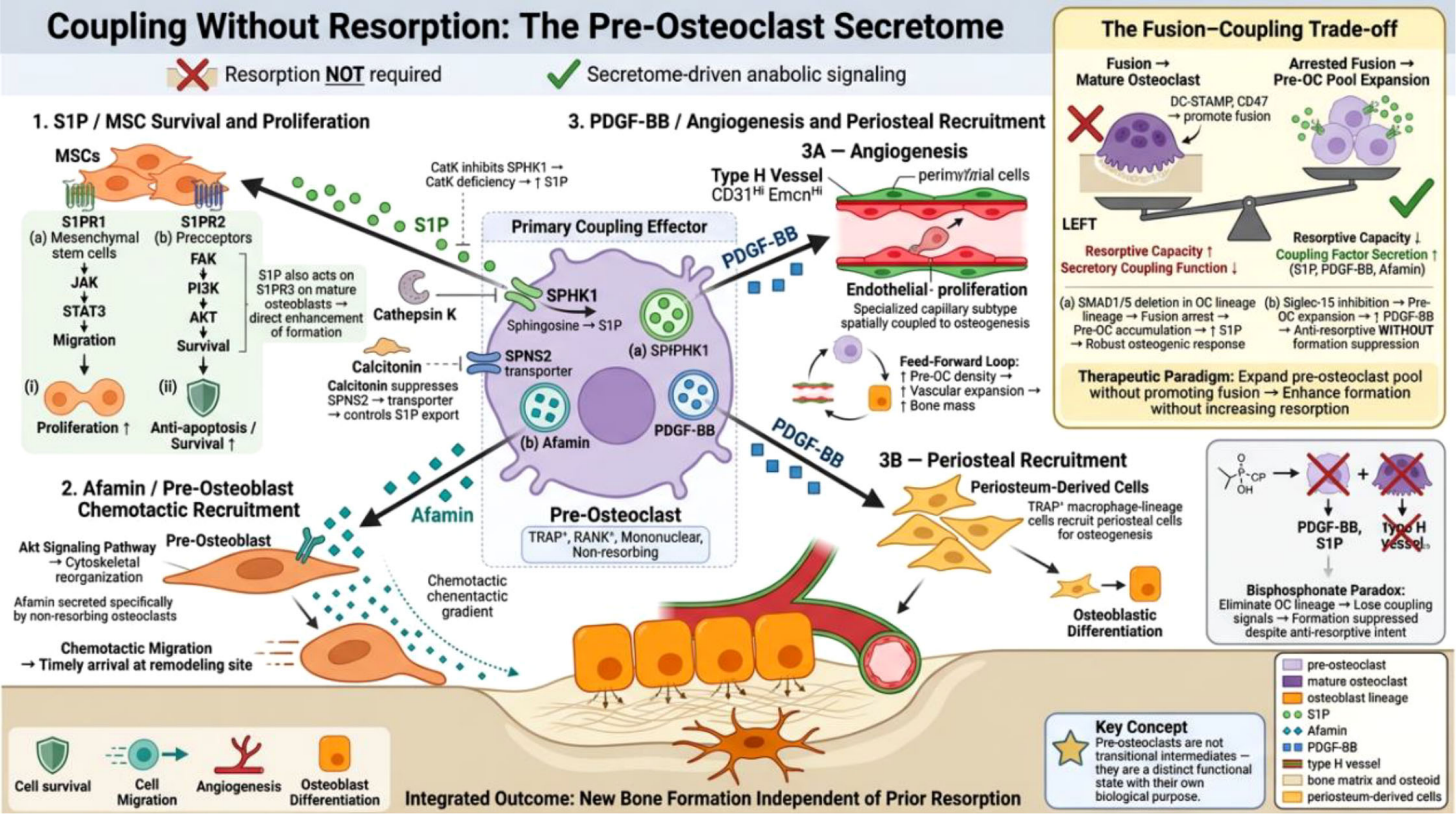
Pre-osteoclasts as primary coupling effectors: the osteo-vascular niche. Pre-osteoclasts (TRAP^+^, mononuclear, non-resorbing) coordinate coupling independently of bone resorption through three secretory pathways: S1P promotes MSC survival via S1PR1/2; afamin directs pre-osteoblast chemotaxis via Akt signaling; and PDGF-BB induces type H vessel formation, establishing a feed-forward loop linking pre-osteoclast density to vascular expansion and bone mass. The inset panel illustrates the fusion–coupling trade-off, where molecular machinery driving fusion antagonizes coupling factor secretion. See Sections 4.1–4.2 for details.

WNT10B extends WNT’s pro-differentiative role to the lineage allocation level, suppressing adipogenesis (C/EBPα, PPARγ) while promoting osteogenic differentiation (Runx2, Osx) (84). The synergistic secretion of WNT10B and S1P by osteoclasts (85), and the pharmacological upregulation of osteoclast-derived WNT10B by agents such as cinacalcet and calcitonin (86, 87), suggest that therapeutic strategies could harness WNT10B’s pro-osteogenic differentiation capacity to redirect MSC fate toward osteogenesis (**Figure 3**).

### 5.4 Context-dependent convergence versus independent operation of coupling cascades

The preceding sections describe BMP, sphingolipid/sclerostin, and WNT signaling as extensively cross-regulated cascades. However, characterizing their interaction as constitutively convergent would be an oversimplification. Whether these pathways operate in a convergent, parallel, or antagonistic manner depends on several context-specific variables that must be explicitly delineated.

During homeostatic remodeling in mechanically loaded, well-vascularized adult bone, convergent signaling predominates. The molecular basis for this convergence is well characterized at specific nodes: BMP-2 facilitates Dvl-1/Smad1 physical interaction, which stabilizes β-catenin and thereby links BMP-driven osteoblast differentiation to Wnt-driven proliferative expansion (70). Simultaneously, S1P secreted by pre-osteoclasts activates S1PR1/2 on MSCs, promoting their migration to remodeling sites where BMP and Wnt ligands are locally concentrated (88). The convergence is further reinforced at the transcriptional level: both BMP-mediated Smad1/5/8 signaling and canonical Wnt/β-catenin signaling converge on Runx2 and Osterix promoter activation, albeit through distinct cis-regulatory elements (71, 72). In this context, the three cascades function as a mutually reinforcing network in which the output of one pathway amplifies the sensitivity or magnitude of the others.

However, at least three categories of conditions favor pathway independence or antagonism. The first is the local concentration of pathway-specific extracellular antagonists. High levels of noggin or gremlin suppress BMP signaling without directly affecting Wnt or S1P pathways (89); elevated DKK1 or sclerostin inhibits canonical Wnt signaling via LRP5/6 without altering BMP receptor activation (76, 77); and altered sphingosine kinase or S1P lyase activity modulates the sphingolipid axis independently of BMP or Wnt ligand availability (90, 91). When one pathway is selectively suppressed by its cognate antagonist while the others remain active, the system transitions from convergent to parallel or compensatory operation. This transition has been observed experimentally: in DKK1-overexpressing transgenic mice, canonical Wnt signaling is profoundly suppressed, yet BMP-mediated osteoblast differentiation persists at reduced efficiency (92), indicating that BMP can drive osteogenesis independently when Wnt input is withdrawn, though the resulting bone formation is quantitatively and qualitatively compromised.

The second is the inflammatory cytokine milieu. TNF-α and IL-1β, which are elevated in inflammatory bone diseases, preferentially amplify RANKL/NF-κB signaling while simultaneously inducing Smurf1-mediated proteasomal degradation of BMP signaling intermediates (93) and upregulating DKK1 expression in osteoblasts and synovial fibroblasts (94). Under these conditions, the sphingolipid axis may shift from its anabolic role toward autocrine regulation of osteoclast precursor trafficking (95), while BMP and Wnt pathways are simultaneously but independently suppressed through distinct molecular mechanisms. The net result is not merely reduced convergent output but a qualitative reconfiguration of the signaling architecture in which pathways that normally reinforce each other become functionally uncoupled.

The third is the differentiation state of the responding cell. Undifferentiated MSCs—which express high levels of S1PR1/2 and Frizzled receptors but relatively low levels of BMPR-IA—are preferentially responsive to S1P chemotactic signals and Wnt-mediated proliferative cues (78, 88). As these cells commit to the osteoblast lineage and upregulate BMPR-IA/IB expression, BMP signaling becomes progressively more influential (63–65), while S1P transitions from a migration signal to a survival factor acting through S1PR3 on committed osteoblasts (88). This differentiation stage–dependent shift in pathway dominance means that the “convergence” observed at the tissue level actually reflects a temporal sequence of pathway engagement at the cellular level, with S1P and Wnt acting primarily on early progenitors and BMP assuming a dominant role during terminal differentiation.

These distinctions carry important implications for therapeutic targeting. Interventions aimed at a single cascade during conditions of pathway convergence may produce network-wide effects through cross-regulation, whereas the same intervention during conditions of pathway independence would produce more circumscribed consequences. This context-dependence helps explain why the skeletal effects of pathway-specific modulators—such as sclerostin antibodies or BMP agonists—vary substantially across clinical populations with different underlying pathophysiology.

### 5.5 Pathological disruption of the coupling signaling network

The context-dependent nature of pathway convergence described above provides a framework for understanding how the coupling network is disrupted in specific bone pathologies. We examine three representative conditions—osteoporosis, Paget’s disease, and metastatic bone disease—that illustrate distinct patterns of network perturbation.

Osteoporosis: progressive erosion of convergent signaling. Postmenopausal osteoporosis exemplifies a condition in which convergent coupling is gradually degraded through multiple concurrent mechanisms. Estrogen withdrawal removes a critical permissive signal for osteoblast progenitor maintenance: ERα signaling normally sustains OPC proliferation and viability while contributing to suppression of osteoclast-derived sclerostin production (43, 44). Its loss simultaneously depletes the target cell population for coupling signals and shifts the S1P-to-sclerostin balance toward inhibition, as discussed in Section 5.2. At the Wnt pathway level, estrogen deficiency has been associated with increased sclerostin and DKK1 expression in osteocytes, directly antagonizing canonical Wnt signaling in osteoblast progenitors (76, 77). Concurrently, the age-related shift in MSC lineage allocation toward adipogenesis—driven by PPARγ activation that suppresses both Runx2 and Wnt/β-catenin signaling (30)—reduces the pool of cells competent to respond to BMP-mediated differentiation signals. The result is a progressive dismantling of the convergent network: each pathway is individually compromised, and the cross-regulatory amplification that normally links them is lost. This framework explains why osteoporosis responds partially to interventions targeting any single pathway—bisphosphonates, romosozumab, or PTH analogs—but cannot be fully reversed by any one approach, as each therapy addresses only one dimension of a multi-pathway failure.

In senile osteoporosis, additional mechanisms compound this network erosion. The age-dependent increase in osteoclast-derived sclerostin (50), combined with declining SPHK1 activity that reduces S1P production, progressively shifts the sphingolipid/sclerostin axis toward net inhibition. Simultaneously, BMP signaling sensitivity may decline due to increased expression of extracellular antagonists including noggin and gremlin in the aging bone marrow microenvironment (89), and reduced BMP receptor density on aged osteoprogenitors. The Wnt pathway is further compromised by age-related accumulation of oxidative stress, which diverts β-catenin from Tcf/Lef-mediated osteogenic transcription toward FoxO-mediated stress response genes, effectively reducing the osteogenic output of Wnt signaling even when ligand levels are maintained (96). This multi-level degradation of all three cascades and their cross-regulatory connections represents a fundamentally different pathological state from early postmenopausal osteoporosis and may require distinct therapeutic strategies that address the network architecture rather than individual pathway nodes.

Paget’s disease: dysregulated amplification rather than suppression. Paget’s disease presents a qualitatively different pattern of network disruption. The hallmark of this condition is excessive, dysregulated osteoclast activity driven by amplified NF-κB signaling, often associated with SQSTM1/p62 mutations that enhance RANK-mediated signaling and, in some cases, paramyxoviral inclusions that further activate NF-κB (97). Unlike osteoporosis, where coupling signals are diminished, Pagetic osteoclasts are characteristically enlarged, hypermultinucleated, and hyperactive, producing supraphysiological levels of coupling factors including TGF-β and IGF-1 released through accelerated resorption (98). However, this excess of coupling signals does not produce normal bone. Instead, the resulting bone is structurally disorganized woven bone with a chaotic lamellar pattern, indicating that the temporal and spatial coordination normally provided by the hierarchical coupling network has been lost.

From the perspective of our framework, Paget’s disease represents a condition in which the quantitative output of individual coupling pathways is elevated but their coordinated regulation is disrupted. The massive, dysregulated release of matrix-derived TGF-β from Pagetic resorption sites may overwhelm the normal BMP signaling hierarchy by saturating Smad2/3 pathways that compete with BMP-activated Smad1/5/8 for nuclear access through the shared co-Smad, Smad4 (99). Furthermore, the excessively rapid turnover rate in Pagetic bone may not allow sufficient time for the sequential pathway engagement described in Section 5.4—S1P-mediated progenitor recruitment followed by Wnt-driven expansion and BMP-mediated terminal differentiation—to proceed in its normal temporal order. The clinical response of Paget’s disease to bisphosphonates, which reduce osteoclast activity and thereby normalize coupling signal output (98), is consistent with this interpretation: restoring normal signal intensity and temporal coordination, rather than enhancing any single pathway, is the therapeutic objective.

Metastatic bone disease: selective hijacking of coupling cascades. Skeletal metastases represent perhaps the most instructive example of how the coupling network can be disrupted, because tumor cells selectively co-opt specific cascades while suppressing others, generating pathological outcomes that cannot be explained by uniform network activation or suppression (100).

In osteolytic metastases, typified by breast cancer bone metastases, tumor-derived PTHrP stimulates RANKL production by osteoblasts and osteocytes, driving excessive osteoclast formation and resorption. This resorption releases matrix-embedded TGF-β, which feeds back to promote further tumor PTHrP secretion—establishing the classical “vicious cycle” (101). Critically, however, tumor cells simultaneously secrete DKK1, which suppresses canonical Wnt signaling in osteoblast progenitors (102), effectively disabling the Wnt-dependent component of the coupling response. The net effect is selective amplification of the resorption-dependent coupling layer (Tier 4 in our framework) while simultaneously suppressing Wnt-dependent components of Tier 1 secreted factor signaling. The BMP pathway may be further compromised by tumor-derived noggin or by epigenetic silencing of BMP receptors in tumor-adjacent osteoblasts (100). This selective pattern of pathway hijacking and suppression explains why osteolytic metastases produce net bone destruction despite the presence of resorption-derived coupling factors: the signals that would normally translate resorption into compensatory formation are specifically disabled by the tumor microenvironment.

In osteoblastic metastases, exemplified by prostate cancer, a reciprocal pattern is observed. Tumor-derived endothelin-1 (103) and BMPs activate osteoblast differentiation, while Wnt pathway modulators including WNT7B may be elevated (104). However, this tumor-driven bone formation is structurally abnormal—mechanically weak woven bone—because it occurs independently of the normal coupling sequence. The osteoblastic response is driven by direct tumor-to-osteoblast signaling rather than by the coordinated, temporally regulated coupling factor release from the osteoclast lineage described in our framework. Mixed metastases, which are common in clinical practice, likely reflect the simultaneous activation of both osteolytic and osteoblastic programs in different microenvironmental niches within the same lesion, consistent with our framework’s prediction that coupling network disruption is spatially heterogeneous.

The therapeutic implications of this disease-specific analysis are significant. In osteoporosis, strategies that restore convergent signaling—by simultaneously enhancing multiple pathway inputs or removing pathway-specific inhibitors—may be more effective than single-pathway interventions. In Paget’s disease, normalizing signal intensity through anti-resorptive therapy is effective precisely because the underlying network architecture remains largely intact. In metastatic bone disease, the selective nature of pathway hijacking suggests that combination therapies targeting the specific pathways exploited by the tumor—rather than broadly modulating coupling—may be required to restore skeletal homeostasis in the metastatic niche.

## 6 Expanding the coupling repertoire: novel factors and contact-dependent signaling

The core cascades described above do not operate in isolation. A growing repertoire of coupling factors—including cardiotrophin-1, SLIT family proteins, complement component C3a, and contact-dependent signaling systems—adds further layers of regulation. The diversity of these mechanisms likely provides the redundancy necessary for such a critical biological process.

However, the evidence supporting each of these factors varies considerably in depth, reproducibility, and translational maturity, and it is essential to delineate these differences explicitly before discussing individual factors. We therefore categorize the coupling factors discussed in this review according to the following evidence hierarchy. The first tier comprises factors for which coupling function has been validated by multiple independent laboratories using *in vivo* genetic models, and for which at least correlative human data exist; S1P, PDGF-BB, and the EPHB4–EFNB2 system fall into this category, supported by converging evidence from conditional knockout mice, pharmacological studies, and clinical biomarker or genetic association data. The second tier includes factors with robust *in vivo* evidence from murine genetic models but limited or absent human clinical validation; cardiotrophin-1 belongs here, as its coupling function is well supported by CT-1-deficient mouse phenotypes but has not been directly examined in human bone biology. The third tier encompasses factors for which *in vivo* evidence exists but is contested between independent research groups, or for which the cellular source remains unresolved; SLIT3 and CTHRC1 fall into this category, as detailed in Sections 6.2 and 8.2, respectively. The fourth tier includes factors whose coupling function has been demonstrated primarily through *in vitro* assays, with limited *in vivo* corroboration; complement component C3a is best placed here, as its osteoblast-stimulatory effects have been shown mainly in cell culture and in a single murine ovariectomy model. Readers should bear this hierarchy in mind when evaluating the therapeutic and translational implications discussed for each factor. We have additionally summarized this evidence classification in **Table 1**.

**Table 1:** Evidence hierarchy for coupling factors discussed in this review.

Coupling factor	Primary model systems	Key *in vivo* evidence	Human clinical data	Evidence tier	Overall confidence
S1P	Murine conditional KO; *in vitro* co-culture	SMAD1/5 deletion, cathepsin K KO, CTR-deficient mice; multiple independent labs	S1P receptor modulators in clinical use (non-skeletal indications); no bone-specific clinical trials	Tier 1	High
PDGF-BB	Murine conditional KO; *in vitro* angiogenesis assays	Pre-osteoclast-specific studies; type H vessel quantification; bisphosphonate suppression models	Correlative: reduced PDGF-BB in aged human bone; no interventional trials	Tier 1	High
EPHB4–EFNB2	Transgenic overexpression; *in vitro* co-culture	EPHB4-overexpressing transgenic mice show increased bone density	No human clinical data	Tier 1–2	High (murine)
CT-1	Global KO mice; *in vitro* signaling studies	CT-1^-^/^-^ mice: reduced trabecular bone, decreased osteoblast numbers	None	Tier 2	Moderate
WNT10B	*In vitro* osteoclast culture; pharmacological modulation	Cinacalcet and calcitonin studies in OVX rats	Indirect: cinacalcet clinical use in CKD patients	Tier 2	Moderate
WNT5A	Osteoclast-specific conditional KO	Wnt5a deletion in osteoclasts reduces bone formation in mice	None	Tier 2	Moderate
SLIT3	Conditional KO (LysM-Cre vs. Ctsk-Cre); BMT; scRNA-seq	Contradictory: severe phenotype with LysM-Cre deletion; no phenotype with Ctsk-Cre deletion	Correlative: plasma LRRD2 levels correlate with BMD in postmenopausal women	Tier 3	Low–Moderate (contested)
CTHRC1	Global KO; osteoblast-specific receptor KO; *in vitro*	Global KO: reduced trabecular bone; cellular source disputed between osteoclast and osteoblast lineages	None	Tier 3	Low (contested source)
C3a	*In vitro* differentiation assays; OVX mouse model	Pharmacological C3aR inhibition exacerbates OVX-induced bone loss (single study)	None	Tier 4	Low
Afamin	*In vitro* chemotaxis assays	Limited *in vivo* validation	None	Tier 4	Low
miR-214-3p (EV)	*In vitro* EV transfer; aged mouse models	Elevated serum exosomal miR-214-3p in aged mice	Correlative: elevated serum exosomal miR-214-3p in osteoporotic patients	Tier 2	Moderate
RANK reverse signaling (EV)	*In vitro* EV co-culture; apoptotic body assays	Limited *in vivo* validation	None	Tier 3–4	Low–Moderate

A specific prediction arising from this layered architecture is that disruption of any single coupling factor should produce only partial coupling failure due to compensatory signaling through parallel mechanisms, whereas simultaneous disruption of factors from different layers—for example, a secreted factor (S1P) combined with a contact-dependent signal (EFNB2)—should produce synergistic rather than additive coupling loss. This prediction can be tested through combinatorial conditional knockout experiments and would, if confirmed, validate the network model over a simple additive-factor model.

### 6.1 Cardiotrophin-1

The coupling factors discussed in Sections 4 and 5 operate primarily by directly stimulating osteoblast recruitment and differentiation. Cardiotrophin-1 (CT-1) adds a mechanistically distinct dimension to this network. Before discussing CT-1’s mechanisms in detail, it is important to note the evidentiary context. CT-1’s classification as an osteoclast-derived coupling factor rests primarily on a single foundational study using global CT-1-knockout mice (105), supplemented by *in vitro* mechanistic work characterizing its signaling through LIFR/gp130 receptor complexes. While the skeletal phenotype of CT-1-deficient mice—reduced trabecular bone and decreased osteoblast numbers—is compelling, several limitations should be acknowledged. The global knockout does not exclude contributions from non-osteoclast sources of CT-1 to the observed phenotype, and osteoclast-lineage-specific conditional deletion of CT-1 has not been reported. Furthermore, no human genetic association studies linking CT-1 variants to bone mineral density or fracture risk have been published, and CT-1 levels have not been systematically measured in human bone biopsy samples or correlated with bone turnover markers in clinical cohorts. The evidence for CT-1 as a coupling factor should therefore be regarded as strong at the murine model level but unvalidated in human systems.

CT-1 was originally identified for its cardiac effects (106) but is expressed in an osteoclast-restricted manner within the skeletal system (107). It signals through LIFR/gp130 heterodimer receptor complexes on osteoblasts, activating STAT3 and ERK cascades that ultimately drive RUNX2-dependent osteocalcin expression through the transcription factor C/EBPδ (105, 108, 109). The skeletal relevance of this pathway is demonstrated by CT-1-deficient mice, which exhibit both reduced trabecular bone parameters and decreased osteoblast numbers, alongside enlarged osteoclasts with compromised resorptive capacity (105). This dual phenotype—affecting both the osteoblast and osteoclast lineages—exemplifies a recurring theme in coupling biology: factors that mediate communication between lineages often regulate both, rather than acting on one in isolation.

What distinguishes CT-1 from many other coupling factors is its capacity to suppress sclerostin expression in osteocytes. As discussed in Section 5.2, the balance between anabolic signals such as S1P and inhibitory signals such as sclerostin is a key determinant of coupling efficiency, and its dysregulation contributes to age-related bone loss. By simultaneously promoting osteoblast differentiation and reducing sclerostin-mediated Wnt inhibition, CT-1 effectively engages both the “accelerator” and “brake” of the coupling system. Additionally, as a member of the gp130 signaling family (110), CT-1 likely integrates with other IL-6 family cytokines in bone regulation, though the details of this integration remain to be fully characterized.

### 6.2 SLIT3: promise and controversy

SLIT family proteins, originally characterized in neuronal guidance (111), have been proposed as skeletal regulators. However, whether SLIT3 functions as a genuine osteoclast-derived coupling factor remains one of the most actively contested questions in the field. The controversy centers on two issues: which cells produce SLIT3 in bone, and through which target cells it exerts its skeletal effects. Importantly, the entire SLIT3 debate has been conducted almost exclusively using murine models—conditional knockout mice, bone marrow transplantation experiments, and murine cell culture systems. Human data are limited to a single correlative observation that plasma levels of SLIT3’s truncated form (LRRD2) correlate with bone mineral density in a cohort of postmenopausal women (112). No human genetic studies have linked SLIT3 variants to skeletal phenotypes, and SLIT3 protein expression has not been systematically characterized in human bone tissue. Given both the unresolved controversy regarding the cellular source in mice and the near-complete absence of human clinical validation, SLIT3’s classification as an osteoclast-derived coupling factor should be regarded as provisional.

Evidence supporting SLIT3 as a coupling factor. Kim et al. first reported that deletion of SLIT3 using LysM-Cre—which targets myeloid lineage cells including osteoclast precursors—produced a dramatic osteoporotic phenotype with reduced bone formation and increased resorption (112). They proposed that SLIT3 secreted by osteoclast-lineage cells acts on ROBO1/2 receptors on osteoblasts, activating β-catenin-dependent proliferation and migration through an Abelson kinase/Cables/cadherin signaling complex (113–115). They further described an autocrine circuit in which SLIT3 signals through ROBO1/3 on osteoclasts themselves, triggering srGAP2-mediated Rac1 suppression to restrain osteoclast activity (116). Xu et al. independently reported skeletal benefits of SLIT3, though their data pointed to a different primary mechanism: stimulation of angiogenesis through endothelial cell migration rather than direct effects on osteoblasts (112, 117). More recently, Kim et al. provided additional evidence showing SLIT3 induction in macrophage-lineage cells via TLR4 activation, with concomitant upregulation of microRNA-218–2 that limits osteoclast commitment (118).

Contradicting evidence. These findings were directly challenged by Li et al., who used Ctsk-Cre-driven conditional deletion—targeting a more mature osteoclast population than LysM-Cre—and found no significant skeletal phenotype in osteoclast-specific SLIT3 knockout mice (119). Through bone marrow transplantation experiments and single-cell RNA sequencing, they further argued that osteoclasts are not a meaningful source of SLIT3 *in vivo* and that SLIT3 expression in bone is primarily attributable to other cell types (119).

Interpreting the discrepancy. The core of this controversy lies in a methodological challenge that extends beyond SLIT3 to the coupling factor field more broadly. LysM-Cre and Ctsk-Cre target overlapping but distinct myeloid populations. LysM-Cre is active in macrophages, dendritic cells, and other myeloid lineages in addition to osteoclast precursors, whereas Ctsk-Cre targets more mature osteoclasts. The divergent phenotypes observed with these two drivers may therefore reflect deletion in different cellular compartments rather than contradictory biology. It remains possible that SLIT3 produced by early myeloid or macrophage-lineage cells—but not by mature osteoclasts—contributes to skeletal homeostasis, which would reconcile both sets of findings. Resolution of this question will likely require more refined Cre drivers with temporal control, combined with spatial transcriptomic approaches that can definitively map SLIT3 expression to specific cell types at active remodeling sites *in vivo*.

### 6.3 Complement Component C3a

The complement system component C3a adds an immune-skeletal interface to the coupling network. However, the evidence supporting C3a as a bona fide osteoclast-derived coupling factor is less extensive than for the factors discussed in Sections 4 and 5, and relies primarily on *in vitro* experiments and a single murine *in vivo* model. Produced by diverse cellular sources including immune cells, liver cells, and osteoclasts (120–123), osteoclast-derived C3a has been shown to enhance osteoblast alkaline phosphatase activity through C3a receptor (C3aR) signaling in cell culture assays (124). The *in vivo* relevance of this pathway is supported by one study demonstrating that pharmacological inhibition of C3aR exacerbates trabecular bone loss and suppresses formation in an ovariectomy-induced bone loss model (124). While this finding is consistent with a coupling function, it should be noted that pharmacological C3aR inhibition affects all C3a signaling systemically—not only osteoclast-derived C3a—and therefore cannot distinguish between osteoclast-specific coupling effects and broader immune-modulatory consequences of complement blockade. No human clinical data directly support a role for osteoclast-derived C3a in bone coupling, and C3a levels have not been correlated with bone turnover markers or bone mineral density in clinical cohorts.

**Figure 4:**
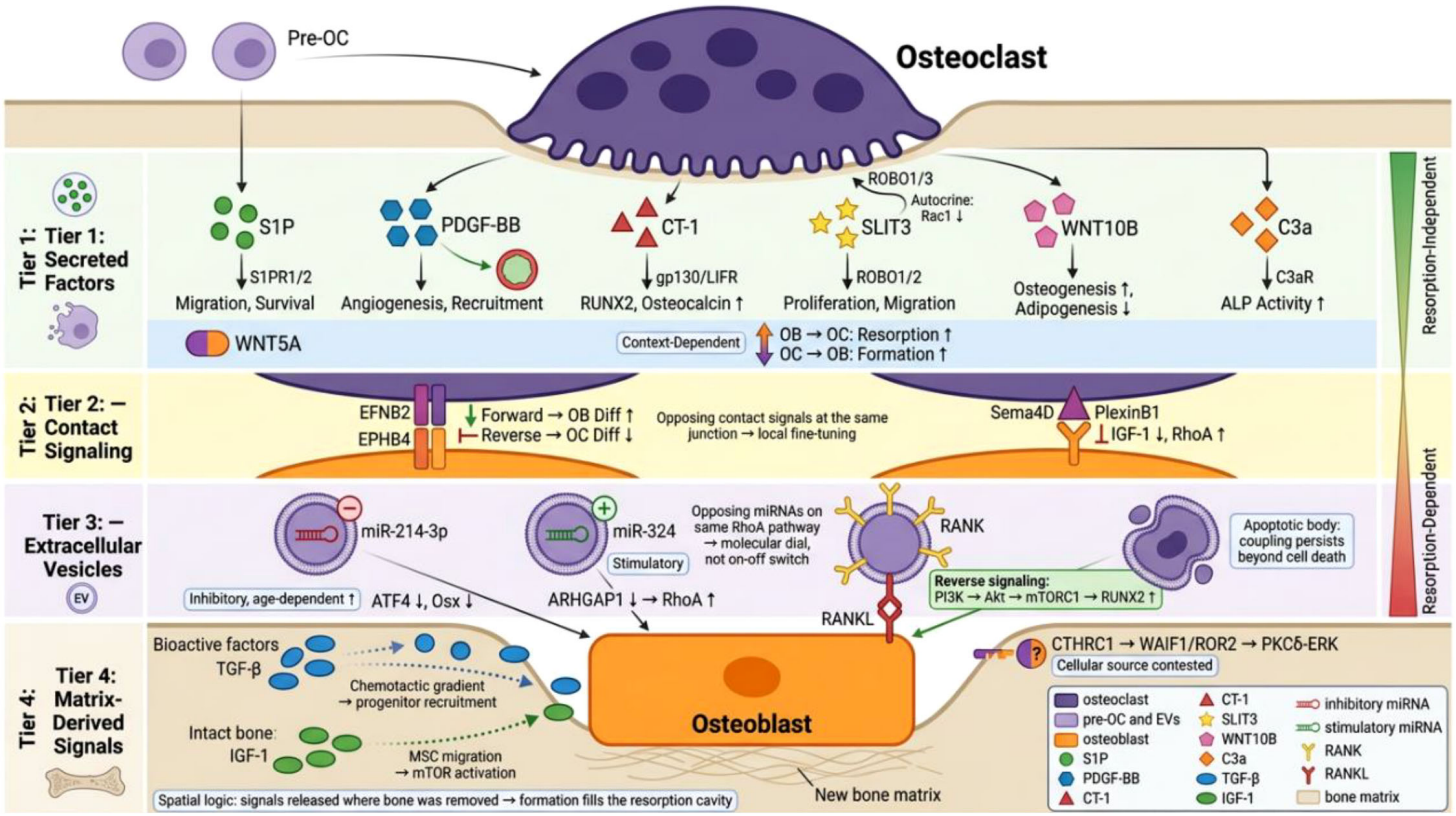
The multi-layered osteoclast–osteoblast communication network. Coupling operates through four interconnected tiers: Tier 1, secreted factors (S1P, PDGF-BB, CT-1, SLIT3, WNT10B, C3a, and context-dependent WNT5A); Tier 2, contact-dependent signaling (EPHB4–EFNB2 and Sema4D–PlexinB1); Tier 3, extracellular vesicles carrying opposing miRNA regulators and RANK for reverse signaling; and Tier 4, matrix-derived signals (TGF-β, IGF-1) released during resorption. The right-margin gradient indicates the spectrum from resorption-independent to resorption-dependent mechanisms. See Sections 5–8 for details.

One notable observation is that while vitamin D induces C3 expression in osteoblasts, these cells appear unable to generate mature C3a protein (124, 125), suggesting that the capacity to produce functional C3a within the bone microenvironment may be preferentially associated with osteoclasts. However, the molecular basis for this difference has not been characterized, and whether this reflects a specific enzymatic processing capability or other factors remains unknown. The involvement of C3a in coupling is nonetheless significant because it links the immune and skeletal systems, raising the possibility that inflammatory conditions that alter complement activation could influence coupling efficiency—a hypothesis that warrants further investigation (**Figure 4**).

### 6.4 Contact-dependent communication: EPH-ephrin and semaphorin systems

Surface-mediated signaling provides contact-dependent bidirectional communication. The EPHB4-EFNB2 system is particularly illustrative: when EFNB2-expressing osteoclasts contact EPHB4-positive osteoblasts, reverse signaling into osteoclasts inhibits differentiation via the c-Fos-NFATc1 axis, while forward signaling promotes osteoblast differentiation and survival through RhoA modulation (126–128). EPHB4 overexpression significantly enhances bone density in transgenic mice.

Semaphorin 4D (Sema4D) provides a complementary membrane-bound signal. Originally identified in neural guidance (129), its interaction with Plexin-B1 on osteoblasts activates RhoA signaling while suppressing IGF-1 pathways (130). These contact-dependent systems have an important property that distinguishes them from secreted factors: they inherently restrict coupling signals to cells in direct physical proximity, providing spatial precision that soluble factors cannot achieve.

### 6.5 S1P receptor-mediated signaling: a versatile coupling mechanism

The S1P axis, introduced earlier in the context of pre-osteoclast biology, merits additional discussion regarding its receptor-level complexity. S1P biosynthesis occurs through SPHK1-catalyzed phosphorylation, with secretion facilitated by the SPNS2 transporter (95, 131). S1P orchestrates bone formation through distinct receptor pathways: S1PR1 and S1PR2 on MSCs activate JAK/STAT3 and FAK/PI3K/AKT cascades to promote migration, while S1PR3 on osteoblasts enhances formation directly (88). S1P also triggers ERK and p38 activation leading to elevated COX2 and PGE2 production, ultimately modulating RANKL expression in osteoblasts (95).

Regulation of this system involves multiple feedback mechanisms. Cathepsin K inhibits SPHK1, while calcitonin suppresses SPNS2 expression (132, 133). CTR-deficient mice exhibit elevated S1P levels and enhanced bone formation (133). Therapeutic S1P analogs have shown promise in promoting bone formation, though the complex interplay between anabolic effects and autocrine osteoclast regulation requires further investigation (90, 91).

## 7 Extracellular vesicle communication: cargo composition, targeting specificity, and matrix-embedded signaling

The signaling mechanisms described above operate through classical paracrine or juxtacrine pathways. Extracellular vesicles (EVs) represent a fundamentally different communication mode, providing protected cargo delivery with enhanced stability and targeting specificity (134, 135). Within the bone microenvironment, osteoclast-derived EVs transfer regulatory molecules—including microRNAs, signaling proteins, and lipid mediators—that add another layer to the coupling network. However, several important caveats must be acknowledged when interpreting the EV literature in bone biology. The majority of functional studies demonstrating EV-mediated osteoclast–osteoblast communication have been performed *in vitro*, often using EV concentrations that may not reflect physiological levels within the bone remodeling compartment. Distinguishing EV-mediated effects from conventional paracrine signaling *in vivo* remains technically challenging, as genetic tools to selectively abolish EV secretion without affecting other cellular functions are limited. Furthermore, EV isolation methods vary substantially across studies, and the heterogeneity of EV preparations—which may contain exosomes, microvesicles, and apoptotic bodies with distinct cargo profiles—complicates cross-study comparisons. These limitations should be considered when evaluating the evidence presented below.

### 7.1 Cargo-loading mechanisms: how osteoclast differentiation state governs vesicular content

A central question in EV-mediated coupling is how the molecular cargo of osteoclast-derived vesicles is determined, and specifically, how the same cell lineage can generate EVs with opposing functional outcomes—promoting or inhibiting osteoblast differentiation—depending on context. The answer lies in the cargo-loading machinery, which is itself regulated by the differentiation state and functional activity of the producing cell.

EV biogenesis in osteoclasts proceeds through at least two major pathways with distinct cargo-sorting properties. The ESCRT (endosomal sorting complexes required for transport)-dependent pathway employs a sequential cascade of ESCRT-0, -I, -II, and -III complexes together with accessory proteins such as ALIX and TSG101 to sort ubiquitinated proteins into intraluminal vesicles within multivesicular bodies (134). This pathway preferentially loads signaling receptors, including RANK, into exosomes—a mechanism directly relevant to the RANK-RANKL reverse signaling axis discussed in Section 7.4. The ESCRT-independent pathway, which relies on ceramide generation by neutral sphingomyelinase 2 (nSMASE2), sorts cargo through lipid raft-mediated mechanisms and preferentially loads certain classes of RNA-binding protein complexes and their associated miRNAs (136). Importantly, the relative activity of these two pathways shifts during osteoclast differentiation: RANKL stimulation upregulates nSMASE2 expression in osteoclast precursors, progressively enriching ceramide-dependent cargo loading as cells mature toward multinucleated osteoclasts. This differentiation-dependent shift in sorting pathway activity provides a molecular mechanism by which the same cell lineage generates EVs with distinct cargo compositions—and therefore distinct functional consequences—at different stages of its maturation.

The specificity of miRNA loading into osteoclast-derived EVs is not random but governed by RNA-binding proteins that recognize specific sequence motifs. Heterogeneous nuclear ribonucleoprotein A2B1 (hnRNPA2B1) recognizes GGAG motifs in miRNA sequences and directs their loading into exosomes following sumoylation-dependent activation (137). Argonaute 2 (Ago2), by contrast, loads a distinct set of miRNAs through RISC complex-dependent mechanisms, and its phosphorylation state—which is regulated by MAPK signaling downstream of RANKL—determines which miRNAs are retained in the cell versus exported in EVs (138). This selective sorting has direct implications for coupling: miR-214-3p, which suppresses osteoblast differentiation, is preferentially loaded through an Ago2-dependent mechanism that is upregulated in mature, actively resorbing osteoclasts (139, 140), whereas miR-324, which promotes osteogenic differentiation, may be sorted through hnRNPA2B1-dependent mechanisms that are more active in earlier osteoclast precursors (141). If confirmed, this sorting-pathway segregation would provide a mechanistic basis for the observation that EVs from pre-osteoclasts versus mature osteoclasts exert different effects on osteoblast differentiation—an extension of the fusion–coupling trade-off described in Section 4.1 to the vesicular communication layer. However, it should be noted that the specific sorting pathways for miR-214-3p and miR-324 in osteoclasts have not been definitively mapped, and this model remains a hypothesis that requires direct experimental validation through RNA-binding protein knockdown studies combined with EV cargo profiling.

Beyond miRNAs, EV protein cargo is also differentiation stage–dependent. Proteomic analyses of EVs from RANKL-stimulated bone marrow macrophages at sequential time points have revealed progressive enrichment of TRAP, cathepsin K fragments, and specific ADAM family metalloproteinases as cells progress from mononuclear precursors to multinucleated osteoclasts, while early-stage EVs are enriched in chemokines and pro-angiogenic factors including VEGF fragments (142). This temporal shift in protein cargo parallels the shift in soluble secretome described for pre-osteoclasts versus mature osteoclasts in Section 4, suggesting that the EV and paracrine communication channels carry complementary but coordinated information about the producing cell’s differentiation state.

### 7.2 Surface determinants of targeting specificity: the molecular address system

The biological impact of an EV depends not only on its cargo but on which target cell it reaches—a property determined by the surface molecule repertoire of both the vesicle and the recipient cell. Osteoclast-derived EVs display a characteristic surface proteome dominated by tetraspanins (CD9, CD63, CD81), integrins (particularly αvβ3), and lineage-specific surface molecules including OSCAR and RANK. These surface molecules function as a “molecular address” that determines targeting specificity through several complementary mechanisms.

Tetraspanin-enriched microdomains on the EV surface organize integrin heterodimers into specific configurations that determine tissue tropism. In the bone microenvironment, αvβ3 integrin on osteoclast-derived EVs engages osteopontin and other RGD-containing matrix proteins deposited on osteoblast surfaces, providing a first layer of targeting selectivity (143). The ephrinA2/EphA2 system provides a second, cell type–specific targeting mechanism: miR-214-3p-containing EVs from mature osteoclasts are enriched in ephrinA2, which engages EphA2 receptors preferentially expressed on osteoblasts, mediating selective uptake through receptor-mediated endocytosis (139, 144). This mechanism ensures that the anti-anabolic miR-214-3p cargo is delivered specifically to osteoblast lineage cells rather than to other cell types in the bone marrow microenvironment—a degree of specificity that free miRNA secretion could not achieve.

Importantly, the EV surface proteome is itself remodeled during osteoclast differentiation. Pre-osteoclast-derived EVs display higher levels of CD9 and lower levels of αvβ3 compared to EVs from mature osteoclasts, and this shift correlates with altered uptake preferences: CD9-high EVs are preferentially internalized by MSCs and early osteoprogenitors, while αvβ3-high EVs are more efficiently taken up by mature osteoblasts (141). If validated by additional studies, this differentiation-dependent surface remodeling would create a system in which EVs from pre-osteoclasts are directed toward early progenitor cells (consistent with their pro-recruitment and pro-proliferative cargo), while EVs from mature osteoclasts target differentiated osteoblasts (consistent with their regulatory and potentially inhibitory cargo). This targeting specificity, combined with the cargo-loading mechanisms described in Section 7.1, would constitute a dual-layer specificity system in which both the message (cargo) and the recipient (surface targeting) are coordinated by the differentiation state of the producing cell.

However, the evidence for differentiation-dependent targeting specificity remains largely correlative, derived from *in vitro* uptake assays using fluorescently labeled EVs. Whether these targeting preferences are maintained in the complex three-dimensional bone marrow microenvironment, where multiple cell types compete for EV uptake and where matrix components may redirect EV tropism, is unknown. Furthermore, most uptake studies cannot distinguish between productive uptake (cargo delivery to the cytoplasm) and non-productive uptake (lysosomal degradation), and the efficiency of cargo delivery *in vivo* remains poorly characterized.

### 7.3 MicroRNA cargo: opposing regulators segregated by sorting machinery

With the cargo-loading and targeting mechanisms established above, the functional consequences of specific miRNA cargo can be better understood. MiR-214-3p, which accumulates in an age-dependent manner in osteoclast-derived EVs, is transferred via the ephrinA2/EphA2-mediated uptake mechanism described in Section 7.2 (139, 140) and suppresses osteoblast differentiation through ATF4 and Osterix inhibition via RhoA activation (139, 144). This pathway has been supported by both *in vitro* and *in vivo* evidence, including elevated serum exosomal miR-214-3p levels in osteoporotic patients and aged mice, making it one of the better-substantiated EV-mediated coupling mechanisms. Conversely, miR-324 has been shown *in vitro* to promote osteogenic differentiation through ARHGAP1 downregulation and subsequent RhoA/ROCK pathway activation (141).

The observation that two EV-associated miRNAs can exert opposing effects on the same RhoA pathway is mechanistically significant. As discussed in Section 7.1, these miRNAs are likely not co-loaded into the same individual vesicles but rather segregated into functionally distinct EV subpopulations through differential RNA-binding protein–dependent sorting. The net effect on osteoblast differentiation at a given remodeling site would therefore depend on the relative abundance of these distinct EV subpopulations—which is in turn determined by the differentiation stage composition of the local osteoclast population. In a remodeling compartment dominated by pre-osteoclasts, the EV milieu would be predicted to favor pro-anabolic miR-324-enriched vesicles; as osteoclasts mature and begin active resorption, the balance would shift toward miR-214-3p-enriched vesicles that restrain osteoblast activity. This model extends the fusion–coupling trade-off from soluble secretory factors to the vesicular communication layer, and predicts that the miR-214-3p/miR-324 ratio in EVs isolated from bone marrow at a given site should correlate with the local ratio of mature to immature osteoclasts—a prediction amenable to testing through combined single-cell analysis and EV profiling from anatomically defined bone compartments. However, this model remains a hypothesis: the two miRNAs have been characterized in separate experimental systems, and direct evidence for their segregation into distinct EV subpopulations from the same osteoclast culture has not been reported.

### 7.4 RANK-RANKL reverse signaling via EVs

One conceptually significant aspect of EV biology in bone is the RANK-RANKL reverse signaling axis (145). The loading of RANK into EVs is mediated through the ESCRT-dependent pathway described in Section 7.1, consistent with the ubiquitination of RANK’s cytoplasmic domain that serves as a sorting signal for ESCRT recognition. Ikebuchi et al. demonstrated that vesicular RANK engages membrane-bound RANKL on osteoblasts, activating PI3K-Akt-mTORC1 cascades and enhancing RUNX2 transcriptional activity (145). The specificity of this interaction is noteworthy: RANK-bearing EVs must engage RANKL in its membrane-anchored trimeric form to activate reverse signaling, and this engagement is facilitated by the orientation of RANK on the EV surface, which preserves the extracellular domain topology necessary for productive RANKL binding. Ma et al. extended this by showing that osteoclast apoptotic bodies retain functional RANK and stimulate mineralization through this mechanism (146), suggesting that the ESCRT-dependent RANK loading that occurs during multivesicular body formation is preserved through the apoptotic process.

These findings are provocative in their implication that osteoclasts may continue to provide coupling signals even during apoptosis. However, several questions remain regarding the physiological significance of this pathway. The quantitative contribution of vesicular RANK reverse signaling relative to other coupling mechanisms has not been established, and whether the concentrations of RANK-bearing EVs generated *in vivo* are sufficient to produce the effects observed *in vitro* is unclear. The hypothesis that residual bone formation observed during aggressive anti-resorptive therapy might be partially maintained by vesicular and apoptotic body–mediated RANK reverse signaling is an interesting possibility that warrants investigation, but remains speculative at present.

### 7.5 Matrixosomes: matrix-embedded vesicles as a temporal memory system

A distinct category of vesicular communication that has received increasing attention is the matrixosome—an extracellular vesicle that becomes embedded within the mineralized bone matrix during osteoblast-mediated bone formation and is subsequently liberated during osteoclastic resorption (147, 148). Matrixosomes represent a fundamentally different temporal logic compared to the freshly secreted EVs discussed above: while conventional EV-mediated signaling operates on timescales of hours to days, matrixosome-mediated signaling introduces a delay of months to years between message encoding (during bone formation) and message delivery (during subsequent resorption), creating a form of molecular memory within the mineralized matrix.

Osteoblast-derived matrixosomes are deposited within the osteoid during matrix synthesis and become entrapped as mineralization proceeds. Their cargo reflects the metabolic and signaling state of the osteoblast at the time of matrix deposition—including specific miRNAs (such as miR-21, miR-29a, and others involved in osteogenic regulation), Wnt ligands, BMPs, and matrix metalloproteinase fragments (147, 148). The mineralized environment selectively preserves certain cargo molecules while degrading others: small RNAs complexed with Ago2 are relatively stable within the mineral matrix, while unprotected mRNAs and certain labile proteins are degraded (149). This selective stabilization means that the cargo delivered when a matrixosome is eventually released during resorption does not perfectly mirror the cargo originally deposited, but rather represents a filtered subset enriched for stable regulatory molecules.

When osteoclasts resorb bone, the acidic environment of the resorption lacuna dissolves the mineral matrix and liberates entrapped matrixosomes. The acidic pH may also modify matrixosome surface molecules, potentially altering their targeting properties compared to freshly secreted EVs (148). Liberated matrixosomes then interact with cells in the local remodeling environment—including osteoblast precursors recruited to fill the resorption cavity—delivering their preserved cargo. This mechanism provides a potential explanation for how the quality of previously formed bone influences the efficiency of subsequent remodeling at the same site: bone formed under healthy conditions would contain matrixosomes with pro-anabolic cargo that supports efficient coupling during future remodeling, while bone formed under pathological conditions (such as during glucocorticoid therapy or in the context of metabolic disease) might contain matrixosomes with altered or depleted cargo that compromises subsequent coupling efficiency.

Several important caveats must be acknowledged regarding the matrixosome concept. First, the distinction between matrixosomes and other matrix-embedded factors (TGF-β, IGF-1) discussed in Section 8.1 is not always clear, and some “matrix-derived” coupling signals previously attributed to free growth factor release may in fact be delivered through matrixosome-mediated mechanisms. Second, the quantitative contribution of matrixosome-delivered cargo relative to freshly secreted EVs and soluble paracrine factors at active remodeling sites has not been established. Third, most evidence for matrixosome biology derives from *in vitro* mineralization and demineralization assays, and the *in vivo* significance of this communication mode requires validation through approaches that can distinguish matrixosome-derived signals from other coupling mechanisms at active remodeling sites. Despite these limitations, the matrixosome concept introduces an important temporal dimension to coupling biology that is not captured by models focused exclusively on contemporaneous cell–cell communication, and it provides a potential mechanistic link between bone quality and coupling efficiency that warrants further investigation.

### 7.6 Engineered EVs and therapeutic potential

The mechanistic understanding of cargo loading and targeting specificity described above has directly informed therapeutic applications (150, 151). Engineered approaches now exploit the natural targeting mechanisms of EVs by modifying surface proteins for enhanced tissue specificity—for example, by displaying bone-targeting peptides (such as alendronate-conjugated or DSPE-PEG-modified surface molecules) that enhance accumulation in the bone microenvironment, or by incorporating specific integrin heterodimers that direct uptake toward defined osteoblast lineage subpopulations. Cargo loading can be manipulated through overexpression of specific RNA-binding proteins to enrich for desired miRNAs, or through electroporation to load exogenous therapeutic molecules.

SPP1-enriched vesicles exemplify the dual functionality possible with EV-based therapeutics, activating TGFβ1/SMAD3 signaling in MSCs while influencing local immune responses, thereby accelerating bone defect repair through coordinated matrix deposition and immunomodulation (152). The understanding that cargo composition determines functional outcome—as established by the sorting mechanisms described in Section 7.1—suggests that therapeutic EVs could be engineered to carry specific combinations of pro-anabolic miRNAs (such as miR-324) while excluding anti-anabolic species (such as miR-214-3p), potentially generating more potent and specific coupling-promoting effects than current approaches. However, manufacturing scalability, cargo standardization, batch-to-batch reproducibility, and regulatory considerations represent substantial barriers to clinical translation that must be addressed before EV-based skeletal therapeutics can advance beyond preclinical proof-of-concept.

## 8 Matrix-derived signals and CTHRC1: the resorption-dependent coupling layer

While pre-osteoclast-derived and vesicle-mediated signals can operate independently of resorption, a distinct set of coupling mechanisms is directly linked to the degradation of mineralized matrix. This resorption-dependent layer ensures that bone formation is stimulated specifically at sites where bone has been removed—providing the spatial precision that is the hallmark of coupled remodeling.

### 8.1 Release of matrix-embedded factors

During resorption, osteoclasts liberate bioactive molecules sequestered within the mineralized matrix (98). TGF-β, complexed with LTBP-3, undergoes activation during this process, initiating cascades that enhance progenitor recruitment and stimulate additional coupling factors including LIF and S1P (153, 154). Matrix-bound IGF-1 complexes similarly become liberated, establishing chemotactic gradients that direct MSC migration to remodeling sites (155, 156). The precision of this system lies in its inherent spatial logic: coupling signals are released precisely where bone has been removed, ensuring that new bone formation fills the resorption cavity.

### 8.2 CTHRC1: a coupling factor with contested origins

Collagen triple helix repeat containing 1 (CTHRC1), originally identified in vascular repair (157), has been proposed as a coupling mediator, though the evidence supporting this classification is less straightforward than for other factors discussed in this review. The evidence for CTHRC1 is notable for being both limited in quantity and internally contradictory. All functional data derive from murine models—global knockout mice, osteoblast-specific receptor deletion, and *in vitro* assays—with no human genetic, clinical, or histological studies directly supporting a coupling role for CTHRC1. Moreover, as detailed below, the two principal studies on CTHRC1 in bone reach conflicting conclusions regarding the cellular source of the protein, creating a level of uncertainty that distinguishes CTHRC1 from better-validated coupling factors. Global genetic ablation of CTHRC1 does produce substantial compromises in trabecular architecture (158), demonstrating that CTHRC1 is important for skeletal homeostasis. CTHRC1 has been shown to signal through WAIF1/ROR2-mediated activation of PKCδ-ERK signaling in osteoblasts (159), and osteoblast-specific WAIF1 deletion reproduces the skeletal phenotypes of CTHRC1 deficiency, confirming the receptor pathway.

However, fundamental questions remain regarding whether CTHRC1 should be classified as an osteoclast-derived coupling factor. First, the primary cellular source is contested. Takeshita et al. reported that CTHRC1 expression correlates with resorptive activity—declining with anti-resorptive interventions and aging—and used osteoclast-conditioned media experiments to support an osteoclast origin (158). In contrast, Jin et al. performed immunohistochemical protein localization studies and found predominant CTHRC1 expression within osteoblast lineage cells rather than osteoclasts (160). Moreover, Jin et al. reported that CTHRC1 actually inhibits osteoclast differentiation *in vitro*, a finding that is difficult to reconcile with its proposed role as an osteoclast-secreted factor and that suggests CTHRC1 may function as an autocrine or paracrine factor within the osteoblast lineage rather than as a coupling signal from osteoclasts to osteoblasts (160).

Second, even if osteoclasts contribute to CTHRC1 production, the correlation between CTHRC1 expression and resorptive activity does not establish a coupling function—it may simply reflect the general metabolic state of the remodeling site. The skeletal phenotype of global CTHRC1 knockout mice, while significant, cannot distinguish between osteoclast-derived, osteoblast-derived, or vascular contributions to the observed bone loss.

This controversy illustrates a broader methodological challenge in coupling factor biology: mRNA expression, protein localization, and functional secretion do not always converge on the same cell type, and the classification of a factor as a “coupling factor” requires not only demonstration of skeletal importance but also evidence that the factor is produced by one lineage and acts on the other in a physiologically meaningful manner. Definitive resolution for CTHRC1 will require cell type–specific conditional knockout approaches in both osteoclast and osteoblast lineages, combined with secretome analysis and *in vivo* validation of the directionality of signaling.

## 9 Therapeutic implications: coupling biology as a framework for understanding treatment limitations and guiding future development

The molecular mechanisms reviewed above provide a framework for understanding one important dimension of why current skeletal therapies, despite their clinical utility, remain fundamentally limited. However, it is essential to recognize at the outset that therapeutic failure in skeletal medicine is multifactorial: conceptual limitations in understanding coupling biology represent only one contributing factor, alongside pharmacological challenges (achieving bone-specific drug exposure), toxicological constraints (off-target effects in non-skeletal tissues), and patient selection complexities (heterogeneity in disease pathophysiology and comorbidity burden) (161, 162). Rather than proposing that coupling factor targeting represents an immediately actionable therapeutic strategy, we argue that an understanding of coupling biology explains certain predictable limitations of existing approaches, while acknowledging that overcoming these limitations will require simultaneous advances across pharmacological, toxicological, and clinical trial design domains. The cases of romosozumab and odanacatib, discussed in detail below, illustrate how these multiple dimensions of translational failure interact in practice.

### 9.1 The anti-resorptive paradox

Bisphosphonates effectively reduce fracture risk by suppressing osteoclast activity and represent a cornerstone of osteoporosis management (163). However, by eliminating osteoclasts and their precursors, these agents simultaneously diminish a source of coupling factors—PDGF-BB, S1P, CT-1, WNT10B, and others—that contribute to driving bone formation (163). This coupling factor loss operates alongside the direct suppression of resorption-dependent matrix-derived signals and potential direct effects of bisphosphonates on osteoblast lineage cells, collectively contributing to the observed decline in bone formation.

It is important to note, however, that the clinical complications associated with prolonged bisphosphonate use—including bisphosphonate-related osteonecrosis of the jaw (BRONJ) and atypical femoral fractures—have multifactorial pathogeneses that cannot be reduced to impaired coupling alone (163, 164). BRONJ involves the convergence of local surgical or infectious insults, impaired mucosal wound healing, altered jawbone vascularity, and immune dysregulation, with suppressed bone turnover serving as a predisposing rather than solely causative factor. Similarly, atypical femoral fractures are primarily attributed to altered bone material properties resulting from profoundly suppressed remodeling turnover, including accumulated microdamage and increased mineralization homogeneity, rather than to coupling factor deficiency perse (163, 164). While coupling disruption likely contributes to the broader decline in bone quality observed during prolonged anti-resorptive therapy—particularly the failure to restore bone mass through compensatory formation—it should be understood as one contributing mechanism within a complex pathological landscape.

The matrix-derived coupling signals TGF-β and IGF-1 are equally disrupted by resorption suppression, as their release requires active resorption (165, 166). The loss of these spatially precise, resorption-site-specific signals may contribute to the inability of bone formation to target sites of accumulated microdamage during prolonged anti-resorptive therapy, though this hypothesis remains to be directly tested.

### 9.2 Sclerostin inhibition: targeted modulation of a specific coupling node

Sclerostin antibodies (romosozumab), approved for the treatment of postmenopausal osteoporosis with high fracture risk and investigated in osteogenesis imperfecta (167–169), target a specific node in the coupling network—osteocyte-derived Wnt inhibition. Their clinical efficacy in reducing vertebral and nonvertebral fracture risk validates the principle that modulating specific coupling pathways can produce meaningful skeletal benefits. However, the clinical trajectory of romosozumab illustrates how pharmacological, toxicological, and patient selection challenges compound the conceptual challenges of targeting coupling pathways.

The cardiovascular safety signal identified in the ARCH trial—an increased incidence of major adverse cardiovascular events compared to alendronate (161)—has been the subject of extensive mechanistic debate. Notably, this signal was not observed in the FRAME trial, which compared romosozumab to placebo (162), raising the possibility that the apparent risk reflects a protective cardiovascular effect of alendronate rather than a harmful effect of romosozumab—a distinction with substantial implications for interpretation. From a pharmacological and toxicological perspective, systemic sclerostin neutralization inevitably modulates Wnt signaling in cardiovascular tissues, where the Wnt pathway regulates vascular calcification, endothelial function, and smooth muscle cell biology (170). Sclerostin is expressed by vascular smooth muscle cells, particularly in calcified atherosclerotic plaques, where it may serve a local protective function by limiting Wnt-driven osteogenic transdifferentiation and vascular calcification; its neutralization could therefore theoretically accelerate vascular pathology in patients with pre-existing atherosclerotic disease (170). However, Mendelian randomization analyses using genetic variants that mimic lifelong sclerostin reduction have yielded inconsistent results regarding cardiovascular risk, suggesting that the relationship between sclerostin inhibition and cardiovascular outcomes may be dose-dependent, duration-dependent, or modified by patient characteristics not captured in genetic instruments (171). From a patient selection perspective, the ARCH trial enrolled women at high fracture risk who inherently carried elevated cardiovascular comorbidity due to shared risk factors including advanced age, systemic inflammation, and metabolic dysfunction (161). Whether the same signal would emerge in a younger or lower-cardiovascular-risk population remains unknown, and the divergent regulatory responses across jurisdictions reflect genuine uncertainty regarding the clinical significance of this finding in different patient populations (172).

The romosozumab experience therefore illustrates a critical principle: even when a coupling-informed therapeutic concept is validated by robust efficacy data, clinical translation can be constrained by off-target pharmacology in non-skeletal tissues and by the inherent difficulty of interpreting safety signals in elderly populations with multiple comorbidities. These challenges are amplified by the fact that most coupling-relevant pathways—including Wnt, BMP, and sphingolipid signaling—have fundamental roles in cardiovascular, immune, and metabolic homeostasis (170).

The observation that CT-1 and LIF suppress sclerostin expression in osteocytes raises the conceptual possibility of achieving sclerostin reduction through endogenous physiological mechanisms rather than exogenous antibody blockade. However, this remains a theoretical consideration: CT-1 and LIF themselves have broad tissue expression and pleiotropic effects that would likely preclude their systemic administration as therapeutics for this purpose.

### 9.3 The cathepsin K lesson: concept validation and translational failure

The cathepsin K inhibitor odanacatib exemplified a conceptually sophisticated therapeutic approach: suppress the resorptive enzyme while preserving osteoclast viability and, by extension, coupling function. Preclinical data supported this concept—cathepsin K-deficient osteoclasts maintained their numbers and exhibited enhanced S1P production through altered SPHK1 activity (132). Phase III clinical trial data from the LOFT study demonstrated significant fracture risk reduction at the hip, spine, and nonvertebral sites, with relatively preserved bone formation markers compared to bisphosphonates—a skeletal profile consistent with the coupling-preservation hypothesis (173). However, drug development was ultimately discontinued due to an increased risk of cerebrovascular events (174, 175).

A thorough analysis of the odanacatib failure requires examination of multiple translational dimensions beyond coupling biology. From a pharmacological perspective, odanacatib required sustained systemic exposure to achieve therapeutic concentrations in the bone remodeling microenvironment, inevitably resulting in cathepsin K inhibition in non-skeletal tissues. From a toxicological perspective, the cerebrovascular signal reflects cathepsin K’s well-characterized role in extracellular matrix remodeling within the vascular wall. Cathepsin K is expressed by macrophages and smooth muscle cells in atherosclerotic plaques, where it participates in collagen and elastin degradation; studies in cathepsin K-deficient mice have demonstrated altered plaque composition with increased fibrosis (176). Inhibition of cathepsin K may therefore alter plaque stability or compromise vascular matrix integrity, potentially increasing cerebrovascular event risk, particularly in the elderly population enrolled in osteoporosis trials. A small but statistically significant increase in morphea-like skin reactions was also observed, reflecting cathepsin K’s role in dermal collagen turnover and further illustrating the breadth of off-target consequences arising from systemic inhibition of a pleiotropic protease (174, 175). From a patient selection perspective, it remains an open question whether the cerebrovascular signal would have been attenuated or absent in a younger population with lower baseline cerebrovascular risk. The LOFT trial enrolled postmenopausal women aged 65 and older, a population with substantial prevalence of subclinical cerebrovascular disease (172). Whether cathepsin K inhibition would have been tolerable in a population selected for low cerebrovascular risk cannot be determined from the available data.

The odanacatib experience provides several instructive lessons that extend beyond the conceptual validation of coupling preservation. First, it demonstrates that favorable skeletal pharmacodynamics—preserved bone formation during resorption suppression—are achievable in human patients, confirming that the fusion–coupling trade-off described in Section 4.1 has clinical relevance. Second, it illustrates that the gap between preclinical concept validation and clinical success is often determined not by the soundness of the biological rationale but by the pharmacological and toxicological properties of the specific drug molecule—properties that are, in principle, addressable through improved drug design or bone-targeted delivery systems (172). Third, it underscores the importance of comprehensive patient stratification: the failure of odanacatib in an unselected elderly population does not necessarily predict failure in a more carefully defined patient population, though this hypothesis remains untested. Cathepsin K, like most molecular participants in coupling, is expressed in multiple tissues, and systemic inhibition produces off-target effects that can outweigh skeletal benefits. This challenge is not unique to cathepsin K but represents a general obstacle for any strategy that seeks to modulate coupling through systemic administration of agents targeting broadly expressed molecules.

### 9.4 Emerging approaches: addressing the bone specificity challenge

Given that the primary translational barrier for coupling-informed therapy is the lack of bone-restricted expression of most coupling factors and their regulators, the most promising emerging approaches are those that attempt to circumvent this limitation through local delivery, targeting of relatively bone-enriched molecules, or microenvironment engineering.

Siglec-15 targeting represents one of the more advanced approaches in this category. Siglec-15 is preferentially expressed on osteoclast lineage cells, providing a degree of tissue selectivity that many other targets lack (60, 61). Anti-Siglec-15 antibodies expand the pre-osteoclast population and enhance PDGF-BB secretion in preclinical models, effectively uncoupling the anti-resorptive effect from formation suppression (60, 61). Phase I/II clinical trial data in postmenopausal osteoporosis have demonstrated increases in bone mineral density with evidence of maintained bone formation markers. However, longer-term efficacy and safety data, including fracture risk reduction, remain to be established.

Bifunctional RANKL antibodies (αR-bif) represent a conceptually distinct approach: simultaneously inhibiting RANKL forward signaling (suppressing osteoclast differentiation) while activating RANKL reverse signaling (stimulating osteoblast function through PI3K-Akt-mTORC1-RUNX2 cascades) (177). This approach has shown efficacy in murine osteoporosis models (177). The observation that sustained bone density improvements during long-term denosumab treatment (178) may partially derive from RANKL reverse signaling activation (179) provides indirect clinical support for this concept, though direct evidence in humans remains limited.

SLIT3’s truncated form (LRRD2) has demonstrated significant improvement in bone parameters in ovariectomized mice while potentially serving as a biomarker, with plasma levels correlating with bone mineral density in postmenopausal women (112). However, given the unresolved controversy regarding the cellular source of SLIT3 discussed in Section 6.2 (112), further validation of this approach is required before it can be considered a viable therapeutic strategy.

Biomaterial-based approaches offer tissue-specific opportunities that bypass the systemic delivery challenge entirely. Titanium nanotubular surfaces have been shown to modulate macrophage and osteoclast secretory profiles in the peri-implant microenvironment, promoting production of osteogenic factors including BMP6, CTHRC1, HGF, SLIT3, and WNT10B (180). This approach—engineering the local microenvironment to enhance endogenous coupling—is most directly applicable to peri-implant bone regeneration and localized bone defect repair rather than systemic skeletal diseases.

Engineered extracellular vesicles offer another localized delivery strategy. SPP1-enriched vesicles have demonstrated efficacy in preclinical bone defect models through coordinated activation of TGFβ1/SMAD3 signaling and immunomodulation (152). The protective cargo delivery afforded by the vesicular format, combined with the potential for surface modification to enhance tissue targeting (150, 151), makes this approach conceptually attractive for localized applications such as fracture nonunion or critical-size defect repair. However, manufacturing scalability, cargo standardization, and regulatory considerations represent substantial barriers to clinical translation.

It is important to acknowledge that none of these emerging approaches have yet demonstrated definitive clinical efficacy in large-scale trials, and significant translational challenges remain for each. The gap between preclinical proof-of-concept and clinical application in coupling biology remains wide, and claims of therapeutic promise should be evaluated against this reality.

**Figure 5:**
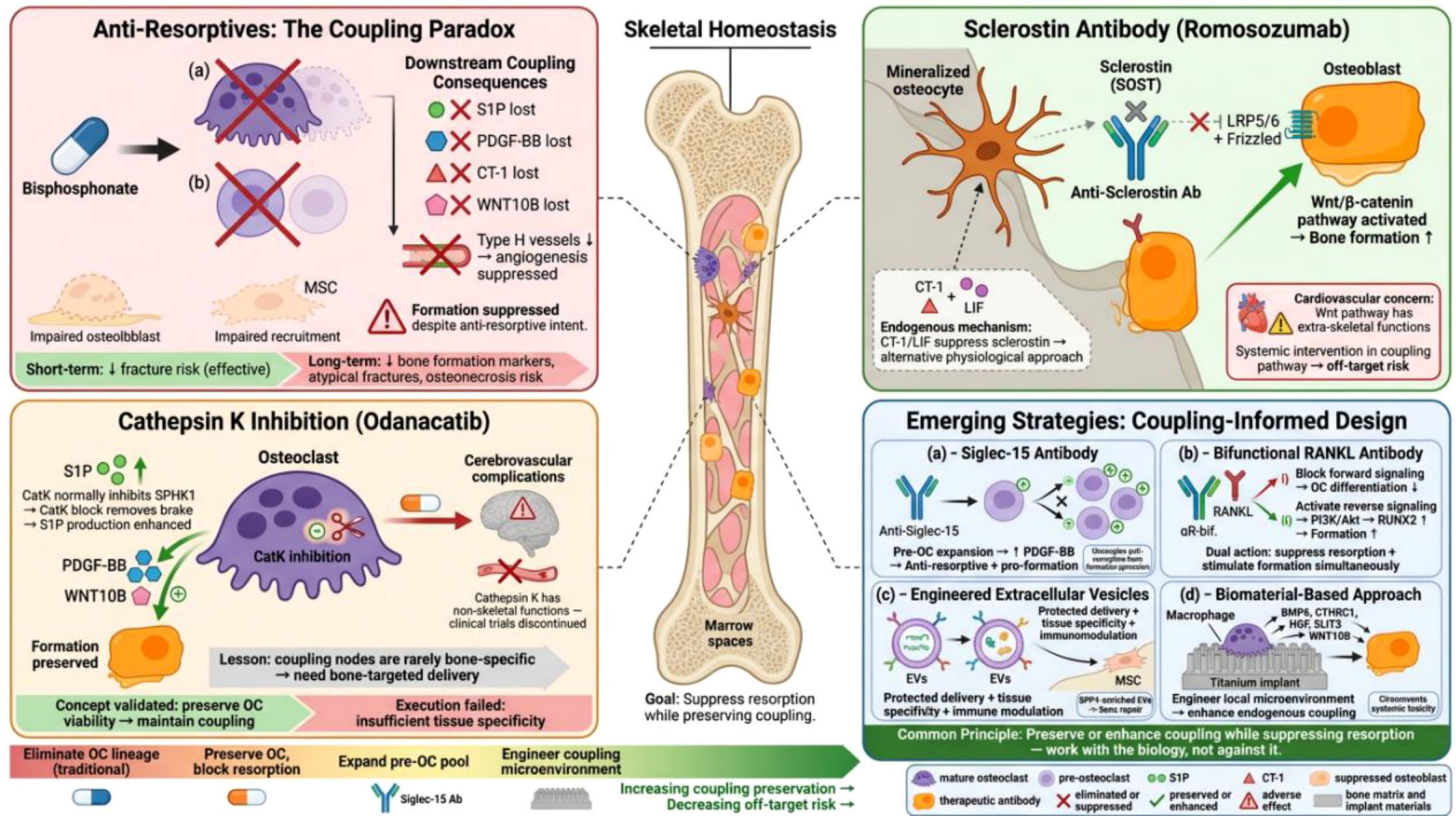
Therapeutic strategies evaluated through the lens of coupling biology. Four therapeutic approaches are compared by their impact on coupling. Bisphosphonates (top-left) eliminate osteoclasts and their coupling secretome, explaining progressive formation decline. Sclerostin antibodies (top-right) target osteocyte-derived Wnt inhibition but carry cardiovascular concerns. Cathepsin K inhibition (bottom-left) preserves osteoclast viability and coupling but was discontinued due to extra-skeletal toxicity. Emerging strategies (bottom-right) include Siglec-15-mediated pre-osteoclast expansion, bifunctional RANKL antibodies, engineered EVs, and biomaterial-based approaches. The bottom bar illustrates the paradigm shift from osteoclast elimination toward coupling preservation. See Section 9 for details.

The observation that coupling factor expression does not necessarily require active resorption—demonstrated in osteopetrotic models and SMAD1/5 deletion studies (181, 182)—is perhaps the most important conceptual insight from the coupling literature for therapeutic development. It establishes the biological principle that the anabolic and resorptive functions of the osteoclast lineage are separable, at least under certain experimental conditions. Whether this separation can be exploited therapeutically in specific clinical contexts—such as postmenopausal osteoporosis, glucocorticoid-induced osteoporosis, or fracture nonunion—remains an open question that will require disease-specific investigation. PTH-based anabolic agents, while effective at promoting formation, trigger compensatory resorption (183), further illustrating the difficulty of selectively engaging one arm of the coupled remodeling process with current pharmacological tools(**Figure 5**).

### 9.5 Integrating multiple dimensions of translational failure

The preceding analysis of individual therapeutic approaches reveals that the persistent difficulty in developing truly disease-modifying skeletal therapies cannot be attributed to any single cause. Rather, translational failure in this field reflects the convergence of at least four distinct but interacting dimensions, and future therapeutic development must address all of them in concert (172).

The first dimension is conceptual: an incomplete understanding of the multi-layered nature of osteoclast–osteoblast coupling has led to therapeutic strategies that inadvertently disrupt the communication networks they seek to preserve. The anti-resorptive paradox (Section 9.1) exemplifies this dimension—bisphosphonates were developed to suppress resorption, and their long-term effects on bone formation were an unanticipated consequence of coupling factor loss (163). The second dimension is pharmacological: achieving bone-restricted drug exposure for agents targeting broadly expressed signaling molecules remains a fundamental unsolved challenge. As illustrated by both romosozumab and odanacatib, systemic administration of agents targeting Wnt pathway components or pleiotropic proteases inevitably produces pharmacological effects in non-skeletal tissues (161, 170, 174, 175). The third dimension is toxicological: the off-target effects that have limited or terminated several promising skeletal therapies reflect the biological reality that most coupling-relevant molecules serve essential functions in cardiovascular, immune, and metabolic systems. The cerebrovascular events with odanacatib and the cardiovascular signal with romosozumab both reflect this reality (161, 176). Importantly, toxicological barriers may be addressable through improved drug design, local delivery strategies such as biomaterial-based approaches and engineered EVs (150, 151), or selection of targets with more restricted expression patterns, with Siglec-15 being a current example (60, 61). The fourth dimension is patient selection: osteoporosis trials typically enroll elderly populations with high comorbidity burden, in whom the risk-benefit ratio of novel therapies may differ fundamentally from that in younger or healthier populations (172). The absence of validated biomarkers that predict individual responses to coupling-modulating therapies—or that identify patients at elevated risk for off-target toxicity—further limits the ability to match patients with optimal treatment strategies.

We therefore emphasize that the coupling biology framework proposed in this review addresses the first of these dimensions and partially informs the second and fourth, but does not by itself resolve the pharmacological and toxicological challenges that have been the proximate cause of most clinical failures. A complete strategy for developing next-generation skeletal therapies must integrate improved conceptual models of coupling biology to identify the most promising targets, innovative pharmacological approaches to achieve bone-specific drug delivery, rigorous toxicological characterization informed by knowledge of target expression across tissues, and sophisticated patient selection strategies guided by coupling-relevant biomarkers (172).

## 10 Conclusions and future perspectives

This review has traced the osteoclast–osteoblast relationship from its developmental origins through to its therapeutic implications, and several overarching principles emerge from this analysis.

The first is that osteoclast heterogeneity is not a developmental artifact but a feature with functional significance. The coexistence of embryonically derived and HSC-derived osteoclast populations throughout life creates a mixed-origin compartment whose coupling properties may differ from either population alone. Testing whether developmental origin influences coupling factor secretion—and whether the ratio of embryonic to HSC-derived osteoclasts changes with aging or disease—should be a priority for future lineage-tracing studies. Critically, as discussed in Section 2.2, this hypothesis has been developed almost exclusively from murine models, and its validation in human systems represents a major unresolved challenge. Potential approaches to bridge this translational gap include single-cell transcriptomic profiling of osteoclast populations isolated from human bone at different skeletal sites and ages, which could reveal whether functionally distinct osteoclast subpopulations exist in humans as they do in mice. Studies of bone remodeling in sex-mismatched bone marrow transplant recipients—where donor-derived and host-derived osteoclasts can be distinguished by sex chromosome analysis—could provide direct evidence regarding the persistence and functional contribution of host-derived (potentially embryonically established) osteoclast populations in the human skeleton. Until such data are available, the ontogeny-coupling framework should be regarded as a hypothesis-generating model rather than an established principle of human skeletal biology.

The second principle is that the pre-osteoclast occupies an important and underappreciated functional position in skeletal biology. By serving as a major source of coupling factors including PDGF-BB, S1P, and afamin, pre-osteoclasts link the osteoclast and osteoblast lineages independently of resorption. The extent to which pre-osteoclast-derived coupling dominates over resorption-dependent mechanisms likely varies by anatomical site, developmental stage, and pathological context—a question that warrants systematic investigation. The fusion–coupling trade-off described here suggests that therapeutic expansion of the pre-osteoclast pool—through SMAD1/5 modulation, Siglec-15 targeting, or other approaches—could represent a genuinely novel therapeutic paradigm.

The third principle is that coupling is mediated not by individual factors but by a multi-layered communication network in which secreted molecules, surface-mediated interactions, extracellular vesicles, and matrix-derived signals operate through shared downstream cascades (BMP, Wnt, sphingolipid). The extensive cross-regulation among these cascades provides robustness but also means that pharmacological intervention at any single node inevitably propagates through the network—explaining why current therapies, however effective at their primary target, always produce secondary effects on coupling.

A fourth principle, prompted by our analysis of therapeutic failures, is that the translational gap in skeletal medicine reflects the convergence of conceptual, pharmacological, toxicological, and patient selection challenges that must be addressed in concert. As the cases of romosozumab and odanacatib demonstrate, even biologically sound therapeutic concepts can fail clinically due to off-target pharmacology, inadequate tissue selectivity, or enrollment of patient populations in whom comorbidity-related risks outweigh skeletal benefits (161, 172). The coupling biology framework proposed here provides a necessary but not sufficient foundation for therapeutic development: it identifies targets and predicts consequences of target modulation, but the realization of coupling-informed therapies will ultimately depend on advances in bone-targeted drug delivery, tissue-selective pharmacology, and biomarker-guided patient stratification that lie outside the scope of coupling biology perse.

We acknowledge that the coupling factor field remains marked by significant unresolved controversies. As discussed in detail above, the cellular source and coupling function of factors including SLIT3 and CTHRC1 are actively debated, and the physiological relevance of EV-mediated communication in bone remodeling *in vivo* requires more rigorous demonstration. The tendency in the literature—and in earlier versions of this review—to present individual coupling factors as confirmed mediators before the evidence has been fully established risks generating a misleading picture of certainty. We have attempted to present these controversies transparently, and we emphasize that the multi-layered framework proposed here should be understood as an evolving model that will require revision as more definitive experimental data emerge.

Throughout this review, we have articulated specific testable predictions arising from each dimension of our framework: origin-dependent coupling factor profiles (Section 2.2), the generalizability of the fusion–coupling trade-off (Section 4.1), age-dependent S1P/sclerostin ratio shifts (Section 5.2), and synergistic coupling failure upon multi-layer disruption (Section 6). An additional prediction with direct translational relevance is that anti-resorptive agents preserving the pre-osteoclast pool should maintain higher long-term bone formation rates than agents eliminating the osteoclast lineage broadly, even at equivalent resorption suppression—a hypothesis testable in head-to-head clinical comparisons using bone formation biomarkers. Collectively, these predictions constitute a research agenda by which the framework can be validated, refined, or refuted; we present them as such, rather than as established conclusions.

Several critical questions remain. First, how does the aging-associated shift in osteoclast secretory profiles—particularly the imbalance between S1P and sclerostin—contribute to the progressive uncoupling of remodeling in senile osteoporosis? Second, can extracellular vesicles be engineered to deliver specific coupling signals with sufficient tissue specificity for clinical application? Third, what mechanisms coordinate the temporal sequence of coupling factor release during the remodeling cycle, and can this temporal code be therapeutically manipulated? Fourth, how do immune-skeletal interfaces—exemplified by the C3a pathway—influence coupling under inflammatory conditions?

Advances in single-cell genomics, spatial transcriptomics, and engineered biomaterials are now providing the tools to address these questions with unprecedented resolution. Success in developing the next generation of skeletal therapies will require moving beyond the reductionist approach of targeting individual resorption or formation pathways, and instead embracing the full complexity of the molecular dialogues that maintain skeletal homeostasis.

## References

[1] Xu F Li W Yang X Na L Chen L Liu G . The roles of epigenetics regulation in bone metabolism and osteoporosis. Front Cell Dev Biol. (2020) 8:619301. doi: 10.3389/fcell.2020.619301. PMID: 33569383 PMC7868402

[2] Letarouilly J-G Broux O Clabaut A . New insights into the epigenetics of osteoporosis. Genomics. (2019) 111:793–8. doi: 10.1016/j.ygeno.2018.05.001. PMID: 29730394

[3] Salari N Ghasemi H Mohammadi L Behzadi MH Rabieenia E Shohaimi S . The global prevalence of osteoporosis in the world: a comprehensive systematic review and meta-analysis. J Orthop Surg Res. (2021) 16:609. doi: 10.1186/s13018-021-02772-0. PMID: 34657598 PMC8522202

[4] Belaya Z Rozhinskaya L Dedov I Drapkina O Fadeev V Golounina O . A summary of the Russian clinical guidelines on the diagnosis and treatment of osteoporosis. Osteoporos Int A J Establ as result Coop between Eur Found Osteoporos Natl Osteoporos Found USA. (2023) 34:429–47. doi: 10.1007/s00198-022-06667-6. PMID: 36651943

[5] Daponte V Henke K Drissi H . Current perspectives on the multiple roles of osteoclasts: mechanisms of osteoclast-osteoblast communication and potential clinical implications. Elife. (2024) 13. doi: 10.7554/eLife.95083. PMID: 38591777 PMC11003748

[6] Fu Y-F Shi S-W Wu J-J Yuan Z-D Wang L-S Nie H . Osteoclast secretes stage-specific key molecules for modulating osteoclast-osteoblast communication. J Cell Physiol. (2025) 240:e31484. doi: 10.1002/jcp.31484. PMID: 39606839

[7] Jiang M Liu R Liu L Kot A Liu X Xiao W . Identification of osteogenic progenitor cell-targeted peptides that augment bone formation. Nat Commun. (2020) 11:4278. doi: 10.1038/s41467-020-17417-9. PMID: 32855388 PMC7453024

[8] Bolamperti S Villa I Rubinacci A . Bone remodeling: an operational process ensuring survival and bone mechanical competence. Bone Res. (2022) 10:48. doi: 10.1038/s41413-022-00219-8. PMID: 35851054 PMC9293977

[9] Munro DAD Hughes J . The origins and functions of tissue-resident macrophages in kidney development. Front Physiol. (2017) 8:837. doi: 10.3389/fphys.2017.00837. PMID: 29118719 PMC5660965

[10] Sheng J Ruedl C Karjalainen K . Most tissue-resident macrophages except microglia are derived from fetal hematopoietic stem cells. Immunity. (2015) 43:382–93. doi: 10.1016/j.immuni.2015.07.016. PMID: 26287683

[11] Hoeffel G Ginhoux F . Fetal monocytes and the origins of tissue-resident macrophages. Cell Immunol. (2018) 330:5–15. doi: 10.1016/j.cellimm.2018.01.001. PMID: 29475558

[12] Tober J Koniski A McGrath KE Vemishetti R Emerson R de Mesy-Bentley KKL . The megakaryocyte lineage originates from hemangioblast precursors and is an integral component both of primitive and of definitive hematopoiesis. Blood. (2007) 109:1433–41. doi: 10.1182/blood-2006-06-031898. PMID: 17062726 PMC1794060

[13] Ginhoux F Greter M Leboeuf M Nandi S See P Gokhan S . Fate mapping analysis reveals that adult microglia derive from primitive macrophages. Science. (2010) 330:841–5. doi: 10.1126/science.1194637. PMID: 20966214 PMC3719181

[14] Italiani P Boraschi D . Development and functional differentiation of tissue-resident versus monocyte-derived macrophages in inflammatory reactions. Results Probl Cell Differ. (2017) 62:23–43. doi: 10.1007/978-3-319-54090-0_2. PMID: 28455704

[15] Gomez Perdiguero E Klapproth K Schulz C Busch K Azzoni E Crozet L . Tissue-resident macrophages originate from yolk-sac-derived erythro-myeloid progenitors. Nature. (2015) 518:547–51. doi: 10.1038/nature13989. PMID: 25470051 PMC5997177

[16] McGrath KE Koniski AD Malik J Palis J . Circulation is established in a stepwise pattern in the mammalian embryo. Blood. (2003) 101:1669–76. doi: 10.1182/blood-2002-08-2531. PMID: 12406884

[17] Kierdorf K Erny D Goldmann T Sander V Schulz C Perdiguero EG . Microglia emerge from erythromyeloid precursors via Pu.1- and Irf8-dependent pathways. Nat Neurosci. (2013) 16:273–80. doi: 10.1038/nn.3318. PMID: 23334579

[18] Jacome-Galarza CE Percin GI Muller JT Mass E Lazarov T Eitler J . Developmental origin, functional maintenance and genetic rescue of osteoclasts. Nature. (2019) 568:541–5. doi: 10.1038/s41586-019-1105-7. PMID: 30971820 PMC6910203

[19] Mass E Ballesteros I Farlik M Halbritter F Günther P Crozet L . Specification of tissue-resident macrophages during organogenesis. Science. (2016) 353. doi: 10.1126/science.aaf4238. PMID: 27492475 PMC5066309

[20] Hoeffel G Chen J Lavin Y Low D Almeida FF See P . C-Myb(+) erythro-myeloid progenitor-derived fetal monocytes give rise to adult tissue-resident macrophages. Immunity. (2015) 42:665–78. doi: 10.1016/j.immuni.2015.03.011. PMID: 25902481 PMC4545768

[21] McGrath KE Frame JM Fegan KH Bowen JR Conway SJ Catherman SC . Distinct sources of hematopoietic progenitors emerge before HSCs and provide functional blood cells in the mammalian embryo. Cell Rep. (2015) 11:1892–904. doi: 10.1016/j.celrep.2015.05.036. PMID: 26095363 PMC4490098

[22] Medvinsky AL Samoylina NL Müller AM Dzierzak EA . An early pre-liver intraembryonic source of CFU-S in the developing mouse. Nature. (1993) 364:64–7. doi: 10.1038/364064a0. PMID: 8316298

[23] Müller AM Medvinsky A Strouboulis J Grosveld F Dzierzak E . Development of hematopoietic stem cell activity in the mouse embryo. Immunity. (1994) 1:291–301. doi: 10.1016/1074-7613(94)90081-7. PMID: 7889417

[24] Yahara Y Barrientos T Tang YJ Puviindran V Nadesan P Zhang H . Erythromyeloid progenitors give rise to a population of osteoclasts that contribute to bone homeostasis and repair. Nat Cell Biol. (2020) 22:49–59. doi: 10.1038/s41556-019-0437-8. PMID: 31907410 PMC6953622

[25] Lee CZW Ginhoux F . Biology of resident tissue macrophages. Development. (2022) 149. doi: 10.1242/dev.200270. PMID: 35502781

[26] Fuller K Owens JM Jagger CJ Wilson A Moss R Chambers TJ . Macrophage colony-stimulating factor stimulates survival and chemotactic behavior in isolated osteoclasts. J Exp Med. (1993) 178:1733–44. doi: 10.1084/jem.178.5.1733. PMID: 8228819 PMC2191238

[27] Zhu S Chen W Masson A Li Y-P . Cell signaling and transcriptional regulation of osteoblast lineage commitment, differentiation, bone formation, and homeostasis. Cell Discov. (2024) 10:71. doi: 10.1038/s41421-024-00689-6. PMID: 38956429 PMC11219878

[28] Ullah I Subbarao RB Rho GJ . Human mesenchymal stem cells - current trends and future prospective. Biosci Rep. (2015) 35. doi: 10.1042/BSR20150025. PMID: 25797907 PMC4413017

[29] Hu L Yin C Zhao F Ali A Ma J Qian A . Mesenchymal stem cells: cell fate decision to osteoblast or adipocyte and application in osteoporosis treatment. Int J Mol Sci. (2018) 19. doi: 10.3390/ijms19020360. PMID: 29370110 PMC5855582

[30] Pino AM Rosen CJ Rodríguez JP . In osteoporosis, differentiation of mesenchymal stem cells (MSCs) improves bone marrow adipogenesis. Biol Res. (2012) 45:279–87. doi: 10.4067/S0716-97602012000300009. PMID: 23283437 PMC8262098

[31] Ambrosi TH Scialdone A Graja A Gohlke S Jank A-M Bocian C . Adipocyte accumulation in the bone marrow during obesity and aging impairs stem cell-based hematopoietic and bone regeneration. Cell Stem Cell. (2017) 20:771–784.e6. doi: 10.1016/j.stem.2017.02.009. PMID: 28330582 PMC5459794

[32] Nahian A Davis DD . Histology, osteoprogenitor cells. In: StatPearls [Internet]. Treasure Island (FL): StatPearls Publishing. (2025). 32644586

[33] Méndez-Ferrer S Michurina TV Ferraro F Mazloom AR Macarthur BD Lira SA . Mesenchymal and haematopoietic stem cells form a unique bone marrow niche. Nature. (2010) 466:829–34. doi: 10.1038/nature09262. PMID: 20703299 PMC3146551

[34] Zhou BO Yue R Murphy MM Peyer JG Morrison SJ . Leptin-receptor-expressing mesenchymal stromal cells represent the main source of bone formed by adult bone marrow. Cell Stem Cell. (2014) 15:154–68. doi: 10.1016/j.stem.2014.06.008. PMID: 24953181 PMC4127103

[35] Worthley DL Churchill M Compton JT Tailor Y Rao M Si Y . Gremlin 1 identifies a skeletal stem cell with bone, cartilage, and reticular stromal potential. Cell. (2015) 160:269–84. doi: 10.1016/j.cell.2014.11.042. PMID: 25594183 PMC4436082

[36] Shi Y He G Lee W-C McKenzie JA Silva MJ Long F . Gli1 identifies osteogenic progenitors for bone formation and fracture repair. Nat Commun. (2017) 8:2043. doi: 10.1038/s41467-017-02171-2. PMID: 29230039 PMC5725597

[37] Park D Spencer JA Koh BI Kobayashi T Fujisaki J Clemens TL . Endogenous bone marrow MSCs are dynamic, fate-restricted participants in bone maintenance and regeneration. Cell Stem Cell. (2012) 10:259–72. doi: 10.1016/j.stem.2012.02.003. PMID: 22385654 PMC3652251

[38] Mizoguchi T Pinho S Ahmed J Kunisaki Y Hanoun M Mendelson A . Osterix marks distinct waves of primitive and definitive stromal progenitors during bone marrow development. Dev Cell. (2014) 29:340–9. doi: 10.1016/j.devcel.2014.03.013. PMID: 24823377 PMC4051418

[39] Debnath S Yallowitz AR McCormick J Lalani S Zhang T Xu R . Discovery of a periosteal stem cell mediating intramembranous bone formation. Nature. (2018) 562:133–9. doi: 10.1038/s41586-018-0554-8. PMID: 30250253 PMC6193396

[40] Kawanami A Matsushita T Chan YY Murakami S . Mice expressing GFP and CreER in osteochondro progenitor cells in the periosteum. Biochem Biophys Res Commun. (2009) 386:477–82. doi: 10.1016/j.bbrc.2009.06.059. PMID: 19538944 PMC2742350

[41] Matthews BG Grcevic D Wang L Hagiwara Y Roguljic H Joshi P . Analysis of αSMA-labeled progenitor cell commitment identifies notch signaling as an important pathway in fracture healing. J Bone Miner Res Off J Am Soc Bone Miner Res. (2014) 29:1283–94. doi: 10.1002/jbmr.2140. PMID: 24190076 PMC4864015

[42] Matthews BG Wee NKY Widjaja VN Price JS Kalajzic I Windahl SH . αSMA osteoprogenitor cells contribute to the increase in osteoblast numbers in response to mechanical loading. Calcif Tissue Int. (2020) 106:208–17. doi: 10.1007/s00223-019-00624-y. PMID: 31673746 PMC6995756

[43] Almeida M Iyer S Martin-Millan M Bartell SM Han L Ambrogini E . Estrogen receptor-α signaling in osteoblast progenitors stimulates cortical bone accrual. J Clin Invest. (2013) 123:394–404. doi: 10.1172/JCI65910. PMID: 23221342 PMC3533305

[44] Syed FA Oursler MJ Hefferanm TE Peterson JM Riggs BL Khosla S . Effects of estrogen therapy on bone marrow adipocytes in postmenopausal osteoporotic women. Osteoporos Int A J Establ as result Coop between Eur Found Osteoporos Natl Osteoporos Found USA. (2008) 19:1323–30. doi: 10.1007/s00198-008-0574-6. PMID: 18274695 PMC2652842

[45] Augustine R Gezek M Nikolopoulos VK Buck PL Bostanci NS Camci-Unal G . Stem cells in bone tissue engineering: progress, promises and challenges. Stem Cell Rev Rep. (2024) 20:1692–731. doi: 10.1007/s12015-024-10738-y. PMID: 39028416 PMC13135464

[46] Ponzetti M Rucci N . Osteoblast differentiation and signaling: established concepts and emerging topics. Int J Mol Sci. (2021) 22. doi: 10.3390/ijms22136651. PMID: 34206294 PMC8268587

[47] Rutkovskiy A Stensløkken K-O Vaage IJ . Osteoblast differentiation at a glance. Med Sci Monit Basic Res. (2016) 22:95–106. doi: 10.12659/msmbr.901142. PMID: 27667570 PMC5040224

[48] Kylmäoja E Nakamura M Turunen S Patlaka C Andersson G Lehenkari P . Peripheral blood monocytes show increased osteoclast differentiation potential compared to bone marrow monocytes. Heliyon. (2018) 4:e00780. doi: 10.1016/j.heliyon.2018.e00780. PMID: 30225379 PMC6138956

[49] Olivera A Spiegel S . Sphingosine-1-phosphate as second messenger in cell proliferation induced by PDGF and FCS mitogens. Nature. (1993) 365:557–60. doi: 10.1038/365557a0. PMID: 8413613

[50] Ota K Quint P Ruan M Pederson L Westendorf JJ Khosla S . Sclerostin is expressed in osteoclasts from aged mice and reduces osteoclast-mediated stimulation of mineralization. J Cell Biochem. (2013) 114:1901–7. doi: 10.1002/jcb.24537. PMID: 23494985 PMC3895454

[51] Zhang Q Long Y Jin L Li C Long J . Non-coding RNAs regulate the BMP/Smad pathway during osteogenic differentiation of stem cells. Acta Histochem. (2023) 125:151998. doi: 10.1016/j.acthis.2023.151998. PMID: 36630753

[52] Tasca A Astleford K Blixt NC Jensen ED Gopalakrishnan R Mansky KC . SMAD1/5 signaling in osteoclasts regulates bone formation via coupling factors. PloS One. (2018) 13:e0203404. doi: 10.1371/journal.pone.0203404. PMID: 30188920 PMC6126839

[53] Ben Shoham A Rot C Stern T Krief S Akiva A Dadosh T . Deposition of collagen type I onto skeletal endothelium reveals a new role for blood vessels in regulating bone morphology. Development. (2016) 143:3933–43. doi: 10.1242/dev.139253. PMID: 27621060 PMC5117144

[54] Zheng Z-W Chen Y-H Wu D-Y Wang J-B Lv M-M Wang X-S . Development of an accurate and proactive immunomodulatory strategy to improve bone substitute material-mediated osteogenesis and angiogenesis. Theranostics. (2018) 8:5482–500. doi: 10.7150/thno.28315. PMID: 30555559 PMC6276091

[55] Kusumbe AP Ramasamy SK Adams RH . Coupling of angiogenesis and osteogenesis by a specific vessel subtype in bone. Nature. (2014) 507:323–8. doi: 10.1038/nature13145. PMID: 24646994 PMC4943525

[56] Kreja L Brenner RE Tautzenberger A Liedert A Friemert B Ehrnthaller C . Non-resorbing osteoclasts induce migration and osteogenic differentiation of mesenchymal stem cells. J Cell Biochem. (2010) 109:347–55. doi: 10.1002/jcb.22406. PMID: 19950208

[57] Xie H Cui Z Wang L Xia Z Hu Y Xian L . PDGF-BB secreted by preosteoclasts induces angiogenesis during coupling with osteogenesis. Nat Med. (2014) 20:1270–8. doi: 10.1038/nm.3668. PMID: 25282358 PMC4224644

[58] Gao B Deng R Chai Y Chen H Hu B Wang X . Macrophage-lineage TRAP+ cells recruit periosteum-derived cells for periosteal osteogenesis and regeneration. J Clin Invest. (2019) 129:2578–94. doi: 10.1172/JCI98857. PMID: 30946695 PMC6538344

[59] Gao S-Y Zheng G-S Wang L Liang Y-J Zhang S-E Lao X-M . Zoledronate suppressed angiogenesis and osteogenesis by inhibiting osteoclasts formation and secretion of PDGF-BB. PloS One. (2017) 12:e0179248. doi: 10.1371/journal.pone.0179248. PMID: 28594896 PMC5464661

[60] Kameda Y Takahata M Komatsu M Mikuni S Hatakeyama S Shimizu T . Siglec-15 regulates osteoclast differentiation by modulating RANKL-induced phosphatidylinositol 3-kinase/Akt and Erk pathways in association with signaling adaptor DAP12. J Bone Miner Res Off J Am Soc Bone Miner Res. (2013) 28:2463–75. doi: 10.1002/jbmr.1989. PMID: 23677868

[61] Zhen G Dan Y Wang R Dou C Guo Q Zarr M . An antibody against Siglec-15 promotes bone formation and fracture healing by increasing TRAP(+) mononuclear cells and PDGF-BB secretion. Bone Res. (2021) 9:47. doi: 10.1038/s41413-021-00161-1. PMID: 34719673 PMC8558327

[62] Kim B-J Lee Y-S Lee S-Y Park S-Y Dieplinger H Ryu SH . Afamin secreted from nonresorbing osteoclasts acts as a chemokine for preosteoblasts via the Akt-signaling pathway. Bone. (2012) 51:431–40. doi: 10.1016/j.bone.2012.06.015. PMID: 22749887

[63] Cao X Chen D . The BMP signaling and *in vivo* bone formation. Gene. (2005) 357:1–8. doi: 10.1016/j.gene.2005.06.017. PMID: 16125875 PMC2667963

[64] Nohno T Ishikawa T Saito T Hosokawa K Noji S Wolsing DH . Identification of a human type II receptor for bone morphogenetic protein-4 that forms differential heteromeric complexes with bone morphogenetic protein type I receptors. J Biol Chem. (1995) 270:22522–6. doi: 10.1074/jbc.270.38.22522. PMID: 7673243

[65] Chen G Deng C Li Y-P . TGF-β and BMP signaling in osteoblast differentiation and bone formation. Int J Biol Sci. (2012) 8:272–88. doi: 10.7150/ijbs.2929. PMID: 22298955 PMC3269610

[66] Anderson HC Hodges PT Aguilera XM Missana L Moylan PE . Bone morphogenetic protein (BMP) localization in developing human and rat growth plate, metaphysis, epiphysis, and articular cartilage. J Histochem Cytochem Off J Histochem Soc. (2000) 48:1493–502. doi: 10.1177/002215540004801106. PMID: 11036092

[67] Peng Y Kang Q Cheng H Li X Sun MH Jiang W . Transcriptional characterization of bone morphogenetic proteins (BMPs)-mediated osteogenic signaling. J Cell Biochem. (2003) 90:1149–65. doi: 10.1002/jcb.10744. PMID: 14635189

[68] Guicheux J Lemonnier J Ghayor C Suzuki A Palmer G Caverzasio J . Activation of p38 mitogen-activated protein kinase and c-Jun-NH2-terminal kinase by BMP-2 and their implication in the stimulation of osteoblastic cell differentiation. J Bone Miner Res Off J Am Soc Bone Miner Res. (2003) 18:2060–8. doi: 10.1359/jbmr.2003.18.11.2060. PMID: 14606520

[69] Lai C-F Cheng S-L . Signal transductions induced by bone morphogenetic protein-2 and transforming growth factor-beta in normal human osteoblastic cells. J Biol Chem. (2002) 277:15514–22. doi: 10.1074/jbc.M200794200. PMID: 11854297

[70] Liu Z Tang Y Qiu T Cao X Clemens TL . A dishevelled-1/Smad1 interaction couples WNT and bone morphogenetic protein signaling pathways in uncommitted bone marrow stromal cells. J Biol Chem. (2006) 281:17156–63. doi: 10.1074/jbc.M513812200. PMID: 16621789

[71] Lee K-S Hong S-H Bae S-C . Both the Smad and p38 MAPK pathways play a crucial role in Runx2 expression following induction by transforming growth factor-beta and bone morphogenetic protein. Oncogene. (2002) 21:7156–63. doi: 10.1038/sj.onc.1205937. PMID: 12370805

[72] Lee M-H Kwon T-G Park H-S Wozney JM Ryoo H-M . BMP-2-induced Osterix expression is mediated by Dlx5 but is independent of Runx2. Biochem Biophys Res Commun. (2003) 309:689–94. doi: 10.1016/j.bbrc.2003.08.058. PMID: 12963046

[73] Dupont S Zacchigna L Cordenonsi M Soligo S Adorno M Rugge M . Germ-layer specification and control of cell growth by Ectodermin, a Smad4 ubiquitin ligase. Cell. (2005) 121:87–99. doi: 10.1016/j.cell.2005.01.033. PMID: 15820681

[74] Datto M Wang X-F . Ubiquitin-mediated degradation a mechanism for fine-tuning TGF-beta signaling. Cell. (2005) 121:2–4. doi: 10.1016/j.cell.2005.03.017. PMID: 15820671

[75] Xu Z Greenblatt MB Yan G Feng H Sun J Lotinun S . SMURF2 regulates bone homeostasis by disrupting SMAD3 interaction with vitamin D receptor in osteoblasts. Nat Commun. (2017) 8:14570. doi: 10.1038/ncomms14570. PMID: 28216630 PMC5321737

[76] Winkler DG Sutherland MK Geoghegan JC Yu C Hayes T Skonier JE . Osteocyte control of bone formation via sclerostin, a novel BMP antagonist. EMBO J. (2003) 22:6267–76. doi: 10.1093/emboj/cdg599. PMID: 14633986 PMC291840

[77] Semënov M Tamai K He X . SOST is a ligand for LRP5/LRP6 and a Wnt signaling inhibitor. J Biol Chem. (2005) 280:26770–5. doi: 10.1074/jbc.M504308200. PMID: 15908424

[78] Kim JH Liu X Wang J Chen X Zhang H Kim SH . Wnt signaling in bone formation and its therapeutic potential for bone diseases. Ther Adv Musculoskelet Dis. (2013) 5:13–31. doi: 10.1177/1759720X12466608. PMID: 23514963 PMC3582304

[79] Albrecht LV Tejeda-Muñoz N De Robertis EM . Cell biology of canonical Wnt signaling. Annu Rev Cell Dev Biol. (2021) 37:369–89. doi: 10.1146/annurev-cellbio-120319-023657. PMID: 34196570

[80] Lojk J Marc J . Roles of non-canonical Wnt signalling pathways in bone biology. Int J Mol Sci. (2021) 22. doi: 10.3390/ijms221910840. PMID: 34639180 PMC8509327

[81] Maeda K Kobayashi Y Udagawa N Uehara S Ishihara A Mizoguchi T . Wnt5a-Ror2 signaling between osteoblast-lineage cells and osteoclast precursors enhances osteoclastogenesis. Nat Med. (2012) 18:405–12. doi: 10.1038/nm.2653. PMID: 22344299

[82] Roberts JL Liu G Paglia DN Kinter CW Fernandes LM Lorenzo J . Deletion of Wnt5a in osteoclasts results in bone loss through decreased bone formation. Ann N Y Acad Sci. (2020) 1463:45–59. doi: 10.1111/nyas.14293. PMID: 31919867

[83] Okamoto M Udagawa N Uehara S Maeda K Yamashita T Nakamichi Y . Noncanonical Wnt5a enhances Wnt/β-catenin signaling during osteoblastogenesis. Sci Rep. (2014) 4:4493. doi: 10.1038/srep04493. PMID: 24670389 PMC3967152

[84] Wend P Wend K Krum SA Miranda-Carboni GA . The role of WNT10B in physiology and disease. Acta Physiol (Oxf). (2012) 204:34–51. doi: 10.1111/j.1748-1716.2011.02296.x. PMID: 21447090

[85] Pederson L Ruan M Westendorf JJ Khosla S Oursler MJ . Regulation of bone formation by osteoclasts involves Wnt/BMP signaling and the chemokine sphingosine-1-phosphate. Proc Natl Acad Sci USA. (2008) 105:20764–9. doi: 10.1073/pnas.0805133106. PMID: 19075223 PMC2603259

[86] Zheng C-M Hsu Y-H Wu C-C Lu C-L Liu W-C Zheng J-Q . Osteoclast-released Wnt-10b underlies cinacalcet related bone improvement in chronic kidney disease. Int J Mol Sci. (2019) 20. doi: 10.3390/ijms20112800. PMID: 31181716 PMC6600662

[87] Hsiao C-Y Chen T-H Chu T-H Ting Y-N Tsai P-J Shyu J-F . Calcitonin induces bone formation by increasing expression of Wnt10b in osteoclasts in ovariectomy-induced osteoporotic rats. Front Endocrinol (Lausanne). (2020) 11:613. doi: 10.3389/fendo.2020.00613. PMID: 33013696 PMC7506163

[88] Quint P Ruan M Pederson L Kassem M Westendorf JJ Khosla S . Sphingosine 1-phosphate (S1P) receptors 1 and 2 coordinately induce mesenchymal cell migration through S1P activation of complementary kinase pathways. J Biol Chem. (2013) 288:5398–406. doi: 10.1074/jbc.M112.413583. PMID: 23300082 PMC3581421

[89] Gazzerro E Canalis E . Bone morphogenetic proteins and their antagonists. Rev Endocr Metab Disord. (2006) 7:51–65. doi: 10.1007/s11154-006-9000-6. PMID: 17029022

[90] Weske S Vaidya M Reese A von Wnuck Lipinski K Keul P Bayer JK . Targeting sphingosine-1-phosphate lyase as an anabolic therapy for bone loss. Nat Med. (2018) 24:667–78. doi: 10.1038/s41591-018-0005-y. PMID: 29662200

[91] Sims NA Martin TJ . Osteoclasts provide coupling signals to osteoblast lineage cells through multiple mechanisms. Annu Rev Physiol. (2020) 82:507–29. doi: 10.1146/annurev-physiol-021119-034425. PMID: 31553686

[92] Li J Sarosi I Cattley RC Pretorius J Asuncion F Grisanti M . Dkk1-mediated inhibition of Wnt signaling in bone results in osteopenia. Bone. (2006) 39:754–66. doi: 10.1016/j.bone.2006.03.017. PMID: 16730481

[93] Guo R Yamashita M Zhang Q Zhou Q Chen D Reynolds DG . Ubiquitin ligase Smurf1 mediates tumor necrosis factor-induced systemic bone loss by promoting proteasomal degradation of bone morphogenetic signaling proteins. J Biol Chem. (2008) 283:23084–92. doi: 10.1074/jbc.M709848200. PMID: 18567580 PMC2517001

[94] Diarra D Stolina M Polzer K Zwerina J Ominsky MS Dwyer D . Dickkopf-1 is a master regulator of joint remodeling. Nat Med. (2007) 13:156–63. doi: 10.1038/nm1538. PMID: 17237793

[95] Ryu J Kim HJ Chang E-J Huang H Banno Y Kim H-H . Sphingosine 1-phosphate as a regulator of osteoclast differentiation and osteoclast-osteoblast coupling. EMBO J. (2006) 25:5840–51. doi: 10.1038/sj.emboj.7601430. PMID: 17124500 PMC1698879

[96] Almeida M Han L Martin-Millan M O’Brien CA Manolagas SC . Oxidative stress antagonizes Wnt signaling in osteoblast precursors by diverting beta-catenin from T cell factor- to forkhead box O-mediated transcription. J Biol Chem. (2007) 282:27298–305. doi: 10.1074/jbc.M702811200. PMID: 17623658

[97] Kravets I . Paget’s disease of bone: Diagnosis and treatment. Am J Med. (2018) 131:1298–303. doi: 10.1016/j.amjmed.2018.04.028. PMID: 29752905

[98] Chen Y Dabovic B Annes JP Rifkin DB . Latent TGF-beta binding protein-3 (LTBP-3) requires binding to TGF-beta for secretion. FEBS Lett. (2002) 517:277–80. doi: 10.1016/s0014-5793(02)02648-0. PMID: 12062452

[99] Massagué J Seoane J Wotton D . Smad transcription factors. Genes Dev. (2005) 19:2783–810. doi: 10.1101/gad.1350705. PMID: 16322555

[100] Weilbaecher KN Guise TA McCauley LK . Cancer to bone: A fatal attraction. Nat Rev Cancer. (2011) 11:411–25. doi: 10.1038/nrc3055. PMID: 21593787 PMC3666847

[101] Yin JJ Selander K Chirgwin JM Dallas M Grubbs BG Wieser R . TGF-beta signaling blockade inhibits PTHrP secretion by breast cancer cells and bone metastases development. J Clin Invest. (1999) 103:197–206. doi: 10.1172/JCI3523. PMID: 9916131 PMC407876

[102] Tian E Zhan F Walker R Rasmussen E Ma Y Barlogie B . The role of the Wnt-signaling antagonist DKK1 in the development of osteolytic lesions in multiple myeloma. N Engl J Med. (2003) 349:2483–94. doi: 10.1056/NEJMoa030847. PMID: 14695408

[103] Nelson JB Hedican SP George DJ Reddi AH Piantadosi S Eisenberger MA . Identification of endothelin-1 in the pathophysiology of metastatic adenocarcinoma of the prostate. Nat Med. (1995) 1:944–9. doi: 10.1038/nm0995-944. PMID: 7585222

[104] Dai J Hall CL Escara-Wilke J Mizokami A Keller JM Keller ET . Prostate cancer induces bone metastasis through Wnt-induced bone morphogenetic protein-dependent and independent mechanisms. Cancer Res. (2008) 68:5785–94. doi: 10.1158/0008-5472.CAN-07-6541. PMID: 18632632 PMC4432935

[105] Walker EC McGregor NE Poulton IJ Pompolo S Allan EH Quinn JMW . Cardiotrophin-1 is an osteoclast-derived stimulus of bone formation required for normal bone remodeling. J Bone Miner Res Off J Am Soc Bone Miner Res. (2008) 23:2025–32. doi: 10.1359/jbmr.080706. PMID: 18665789

[106] Pennica D King KL Shaw KJ Luis E Rullamas J Luoh SM . Expression cloning of cardiotrophin 1, a cytokine that induces cardiac myocyte hypertrophy. Proc Natl Acad Sci USA. (1995) 92:1142–56. doi: 10.1073/pnas.92.4.1142. PMID: 7862649 PMC42654

[107] Sims NA Walsh NC . GP130 cytokines and bone remodelling in health and disease. BMB Rep. (2010) 43:513–23. doi: 10.5483/bmbrep.2010.43.8.513. PMID: 20797312

[108] Liu NQ Lin Y Li L Lu J Geng D Zhang J . gp130/STAT3 signaling is required for homeostatic proliferation and anabolism in postnatal growth plate and articular chondrocytes. Commun Biol. (2022) 5:64. doi: 10.1038/s42003-021-02944-y. PMID: 35039652 PMC8763901

[109] Johnson RW McGregor NE Brennan HJ Crimeen-Irwin B Poulton IJ Martin TJ . Glycoprotein130 (Gp130)/interleukin-6 (IL-6) signalling in osteoclasts promotes bone formation in periosteal and trabecular bone. Bone. (2015) 81:343–51. doi: 10.1016/j.bone.2015.08.005. PMID: 26255596

[110] López-Yoldi M Moreno-Aliaga MJ Bustos M . Cardiotrophin-1: A multifaceted cytokine. Cytokine Growth Factor Rev. (2015) 26:523–32. doi: 10.1016/j.cytogfr.2015.07.009. PMID: 26188636

[111] Dou C Wang H Zhou G Zhu H Wen H Xu S . Slit3 regulates migration of endothelial progenitor cells by activation of the RhoA/Rho kinase pathway. Int J Clin Exp Pathol. (2018) 11:3398–404. PMC6962882 31949717

[112] Kim BJ Lee YS Lee SY Baek WY Choi YJ Moon SA . Osteoclast-secreted SLIT3 coordinates bone resorption and formation. J Clin Invest. (2018) 128:1429–41. doi: 10.1172/JCI91086. PMID: 29504949 PMC5873876

[113] Blockus H Chédotal A . Slit-robo signaling. Development. (2016) 143:3037–44. doi: 10.1242/dev.132829. PMID: 27578174

[114] Rhee J Buchan T Zukerberg L Lilien J Balsamo J . Cables links Robo-bound Abl kinase to N-cadherin-bound beta-catenin to mediate Slit-induced modulation of adhesion and transcription. Nat Cell Biol. (2007) 9:883–92. doi: 10.1038/ncb1614. PMID: 17618275

[115] Koohini Z Koohini Z Teimourian S . Slit/Robo signaling pathway in cancer; a new stand point for cancer treatment. Pathol Oncol Res. (2019) 25:1285–93. doi: 10.1007/s12253-018-00568-y. PMID: 30610466

[116] Shin B Kupferman J Schmidt E Polleux F Delany AM Lee SK . Rac1 inhibition via Srgap2 restrains inflammatory osteoclastogenesis and limits the clastokine, SLIT3. J Bone Miner Res Off J Am Soc Bone Miner Res. (2020) 35:789–800. doi: 10.1002/jbmr.3945. PMID: 31880824 PMC7690287

[117] Xu R Yallowitz A Qin A Wu Z Shin DY Kim JM . Targeting skeletal endothelium to ameliorate bone loss. Nat Med. (2018) 24:823–33. doi: 10.1038/s41591-018-0020-z. PMID: 29785024 PMC5992080

[118] Kim EY Kim JE Chung SH Park JE Yoon D Min HJ . Concomitant induction of SLIT3 and microRNA-218–2 in macrophages by toll-like receptor 4 activation limits osteoclast commitment. Cell Commun Signal. (2023) 21:213. doi: 10.1186/s12964-023-01226-w. PMID: 37596575 PMC10436635

[119] Li N Inoue K Sun J Niu Y Lalani S Yallowitz A . Osteoclasts are not a source of SLIT3. Bone Res. (2020) 8:11. doi: 10.1038/s41413-020-0086-3. PMID: 32133214 PMC7031526

[120] Dunkelberger JR Song WC . Complement and its role in innate and adaptive immune responses. Cell Res. (2010) 20:34–50. doi: 10.1038/cr.2009.139. PMID: 20010915

[121] Lubbers R van Essen MF van Kooten C Trouw LA . Production of complement components by cells of the immune system. Clin Exp Immunol. (2017) 188:183–94. doi: 10.1111/cei.12952. PMID: 28249350 PMC5383442

[122] Han J Zhang X . Complement component C3: A novel biomarker participating in the pathogenesis of non-alcoholic fatty liver disease. Front Med. (2021) 8:653293. doi: 10.3389/fmed.2021.653293. PMID: 34395461 PMC8358116

[123] Ignatius A Schoengraf P Kreja L Liedert A Recknagel S Kandert S . Complement C3a and C5a modulate osteoclast formation and inflammatory response of osteoblasts in synergism with IL-1β. J Cell Biochem. (2011) 112:2594–605. doi: 10.1002/jcb.23186. PMID: 21598302 PMC3158833

[124] Matsuoka K Park KA Ito M Ikeda K Takeshita S . Osteoclast-derived complement component 3a stimulates osteoblast differentiation. J Bone Miner Res Off J Am Soc Bone Miner Res. (2014) 29:1522–30. doi: 10.1002/jbmr.2187. PMID: 24470120

[125] Sato T Hong MH Jin CH Ishimi Y Udagawa N Shinki T . The specific production of the third component of complement by osteoblastic cells treated with 1 alpha,25-dihydroxyvitamin D3. FEBS Lett. (1991) 285:21–4. doi: 10.1016/0014-5793(91)80715-f. PMID: 2065778

[126] Tonna S Takyar FM Vrahnas C Crimeen-Irwin B Ho PWM Poulton IJ . EphrinB2 signaling in osteoblasts promotes bone mineralization by preventing apoptosis. FASEB J Off Publ Fed Am Soc Exp Biol. (2014) 28:4482–96. doi: 10.1096/fj.14-254300. PMID: 24982128

[127] Pasquale EB . Eph receptors and ephrins in cancer: bidirectional signalling and beyond. Nat Rev Cancer. (2010) 10:165–80. doi: 10.1038/nrc2806. PMID: 20179713 PMC2921274

[128] Zhao C Irie N Takada Y Shimoda K Miyamoto T Nishiwaki T . Bidirectional ephrinB2-EphB4 signaling controls bone homeostasis. Cell Metab. (2006) 4:111–21. doi: 10.1016/j.cmet.2006.05.012. PMID: 16890539

[129] Hall KT Boumsell L Schultze JL Boussiotis VA Dorfman DM Cardoso AA . Human CD100, a novel leukocyte semaphorin that promotes B-cell aggregation and differentiation. Proc Natl Acad Sci USA. (1996) 93:11780–5. doi: 10.1073/pnas.93.21.11780. PMID: 8876214 PMC38135

[130] Negishi-Koga T Shinohara M Komatsu N Bito H Kodama T Friedel RH . Suppression of bone formation by osteoclastic expression of semaphorin 4D. Nat Med. (2011) 17:1473–80. doi: 10.1038/nm.2489. PMID: 22019888

[131] Mendelson K Evans T Hla T . Sphingosine 1-phosphate signalling. Development. (2014) 141:5–9. doi: 10.1242/dev.094805. PMID: 24346695 PMC3865745

[132] Lotinun S Kiviranta R Matsubara T Alzate JA Neff L Lüth A . Osteoclast-specific cathepsin K deletion stimulates S1P-dependent bone formation. J Clin Invest. (2013) 123:666–81. doi: 10.1172/JCI64840. PMID: 23321671 PMC3561821

[133] Keller J Catala-Lehnen P Huebner AK Jeschke A Heckt T Lueth A . Calcitonin controls bone formation by inhibiting the release of sphingosine 1-phosphate from osteoclasts. Nat Commun. (2014) 5:5215. doi: 10.1038/ncomms6215. PMID: 25333900 PMC4205484

[134] Colombo M Raposo G Théry C . Biogenesis, secretion, and intercellular interactions of exosomes and other extracellular vesicles. Annu Rev Cell Dev Biol. (2014) 30:255–89. doi: 10.1146/annurev-cellbio-101512-122326. PMID: 25288114

[135] Liu M Sun Y Zhang Q . Emerging role of extracellular vesicles in bone remodeling. J Dent Res. (2018) 97:859–68. doi: 10.1177/0022034518764411. PMID: 29566346

[136] Trajkovic K Hsu C Chiantia S Rajendran L Wenzel D Wieland F . Ceramide triggers budding of exosome vesicles into multivesicular endosomes. Science. (2008) 319:1244–7. doi: 10.1126/science.1153124. PMID: 18309083

[137] Villarroya-Beltri C Gutiérrez-Vázquez C Sánchez-Cabo F Pérez-Hernández D Vázquez J Martin-Cofreces N . Sumoylated hnRNPA2B1 controls the sorting of miRNAs into exosomes through binding to specific motifs. Nat Commun. (2013) 4:2980. doi: 10.1038/ncomms3980. PMID: 24356509 PMC3905700

[138] McKenzie AJ Hoshino D Hong NH Cha DJ Franklin JL Coffey RJ . KRAS-MEK signaling controls Ago2 sorting into exosomes. Cell Rep. (2016) 15:978–87. doi: 10.1016/j.celrep.2016.03.085. PMID: 27117408 PMC4857875

[139] Sun W Zhao C Li Y Wang L Nie G Peng J . Osteoclast-derived microRNA-containing exosomes selectively inhibit osteoblast activity. Cell Discov. (2016) 2:16015. doi: 10.1038/celldisc.2016.15. PMID: 27462462 PMC4886818

[140] Li D Liu J Guo B Liang C Dang L Lu C . Osteoclast-derived exosomal miR-214-3p inhibits osteoblastic bone formation. Nat Commun. (2016) 7:10872. doi: 10.1038/ncomms10872. PMID: 26947250 PMC4786676

[141] Liang M Yin X Zhang S Ai H Luo F Xu J . Osteoclast-derived small extracellular vesicles induce osteogenic differentiation via inhibiting ARHGAP1. Mol Ther Nucleic Acids. (2021) 23:1191–203. doi: 10.1016/j.omtn.2021.01.031. PMID: 33664997 PMC7900016

[142] Patil KC Soekmadji C . Extracellular vesicle-mediated bone remodeling and bone metastasis: Implications in prostate cancer. Sub-cell Biochem. (2021) 97:297–361. doi: 10.1007/978-3-030-67171-6_12. PMID: 33779922

[143] Huynh N VonMoss L Smith D Rahman I Felemban MF Zuo J . Characterization of regulatory extracellular vesicles from osteoclasts. J Dent Res. (2016) 95:673–9. doi: 10.1177/0022034516633189. PMID: 26908631 PMC4924543

[144] Wang X Guo B Li Q Peng J Yang Z Wang A . miR-214 targets ATF4 to inhibit bone formation. Nat Med. (2013) 19:93–100. doi: 10.1038/nm.3026. PMID: 23223004

[145] Ikebuchi Y Aoki S Honma M Hayashi M Sugamori Y Khan M . Coupling of bone resorption and formation by RANKL reverse signalling. Nature. (2018) 561:195–200. doi: 10.1038/s41586-018-0482-7. PMID: 30185903

[146] Ma Q Liang M Wu Y Luo F Ma Z Dong S . Osteoclast-derived apoptotic bodies couple bone resorption and formation in bone remodeling. Bone Res. (2021) 9:5. doi: 10.1038/s41413-020-00121-1. PMID: 33431863 PMC7801485

[147] Davies OG Cox SC Azoidis I McGuinness AJA Cooke M Heaney LM . Osteoblast-derived vesicle protein content is temporally regulated during osteogenesis: Implications for regenerative therapies. Front Bioeng Biotechnol. (2019) 7:92. doi: 10.3389/fbioe.2019.00092. PMID: 31119130 PMC6504811

[148] Morhayim J van de Peppel J Demmers JAA Kocer G Nigg AL van Driel M . Proteomic signatures of extracellular vesicles secreted by nonmineralizing and mineralizing human osteoblasts and stimulation of tumor cell growth. FASEB J Off Publ Fed Am Soc Exp Biol. (2015) 29:274–85. doi: 10.1096/fj.14-261404. PMID: 25359493

[149] Mateescu B Kowal EJK van Balkom BWM Bartel S Bhattacharyya SN Buzás EI . Obstacles and opportunities in the functional analysis of extracellular vesicle RNA - an ISEV position paper. J Extracell Vesicles. (2017) 6:1286095. doi: 10.1080/20013078.2017.1286095. PMID: 28326170 PMC5345583

[150] Lo Cicero A Stahl PD Raposo G . Extracellular vesicles shuffling intercellular messages: for good or for bad. Curr Opin Cell Biol. (2015) 35:69–77. doi: 10.1016/j.ceb.2015.04.013. PMID: 26001269

[151] Yáñez-Mó M Siljander P-M Andreu Z Zavec AB Borràs FE Buzas EI . Biological properties of extracellular vesicles and their physiological functions. J Extracell Vesicles. (2015) 4:27066. doi: 10.3402/jev.v4.27066. PMID: 25979354 PMC4433489

[152] Faqeer A Wang M Alam G Padhiar AA Zheng D Luo Z . Cleaved SPP1-rich extracellular vesicles from osteoclasts promote bone regeneration via TGFβ1/SMAD3 signaling. Biomaterials. (2023) 303:122367. doi: 10.1016/j.biomaterials.2023.122367. PMID: 38465579

[153] Ota K Quint P Weivoda MM Ruan M Pederson L Westendorf JJ . Transforming growth factor beta 1 induces CXCL16 and leukemia inhibitory factor expression in osteoclasts to modulate migration of osteoblast progenitors. Bone. (2013) 57:68–75. doi: 10.1016/j.bone.2013.07.023. PMID: 23891907 PMC3845829

[154] Centrella M Massagué J Canalis E . Human platelet-derived transforming growth factor-beta stimulates parameters of bone growth in fetal rat calvariae. Endocrinology. (1986) 119:2306–12. doi: 10.1210/endo-119-5-2306. PMID: 3464414

[155] Xian L Wu X Pang L Lou M Rosen CJ Qiu T . Matrix IGF-1 maintains bone mass by activation of mTOR in mesenchymal stem cells. Nat Med. (2012) 18:1095–101. doi: 10.1038/nm.2793. PMID: 22729283 PMC3438316

[156] Tang Y Wu X Lei W Pang L Wan C Shi Z . TGF-beta1-induced migration of bone mesenchymal stem cells couples bone resorption with formation. Nat Med. (2009) 15:757–65. doi: 10.1038/nm.1979. PMID: 19584867 PMC2727637

[157] Pyagay P Heroult M Wang Q Lehnert W Belden J Liaw L . Collagen triple helix repeat containing 1, a novel secreted protein in injured and diseased arteries, inhibits collagen expression and promotes cell migration. Circ Res. (2005) 96:261–8. doi: 10.1161/01.RES.0000154262.07264.12. PMID: 15618538

[158] Takeshita S Fumoto T Matsuoka K Park K Aburatani H Kato S . Osteoclast-secreted CTHRC1 in the coupling of bone resorption to formation. J Clin Invest. (2013) 123:3914–24. doi: 10.1172/JCI69493. PMID: 23908115 PMC3754269

[159] Matsuoka K Kohara Y Naoe Y Watanabe A Ito M Ikeda K . WAIF1 is a cell-surface CTHRC1 binding protein coupling bone resorption and formation. J Bone Miner Res Off J Am Soc Bone Miner Res. (2018) 33:1500–12. doi: 10.1002/jbmr.3436. PMID: 29624737

[160] Jin Y-R Stohn JP Wang Q Nagano K Baron R Bouxsein ML . Inhibition of osteoclast differentiation and collagen antibody-induced arthritis by CTHRC1. Bone. (2017) 97:153–67. doi: 10.1016/j.bone.2017.01.022. PMID: 28115279 PMC6746321

[161] Saag KG Petersen J Brandi ML Karaplis AC Lorentzon M Thomas T . Romosozumab or alendronate for fracture prevention in women with osteoporosis. N Engl J Med. (2017) 377:1417–27. doi: 10.1056/NEJMoa1708322. PMID: 28892457

[162] Cosman F Crittenden DB Adachi JD Binkley N Czerwinski E Ferrari S . Romosozumab treatment in postmenopausal women with osteoporosis. N Engl J Med. (2016) 375:1532–43. doi: 10.1056/NEJMoa1607948. PMID: 27641143

[163] Reyes C Hitz M Prieto-Alhambra D Abrahamsen B . Risks and benefits of bisphosphonate therapies. J Cell Biochem. (2016) 117:20–8. doi: 10.1002/jcb.25266. PMID: 26096687

[164] Koh JSB Goh SK Png MA Kwek EBK Howe TS . Femoral cortical stress lesions in long-term bisphosphonate therapy: a herald of impending fracture? J Orthop Trauma. (2010) 24:75–81. doi: 10.1097/BOT.0b013e3181b6499b. PMID: 20101130

[165] Wang Y Nishida S Elalieh HZ Long RK Halloran BP Bikle DD . Role of IGF-I signaling in regulating osteoclastogenesis. J Bone Miner Res Off J Am Soc Bone Miner Res. (2006) 21:1350–8. doi: 10.1359/jbmr.060610. PMID: 16939393 PMC10723110

[166] Reijnders CMA Bravenboer N Tromp AM Blankenstein MA Lips P . Effect of mechanical loading on insulin-like growth factor-I gene expression in rat tibia. J Endocrinol. (2007) 192:131–40. doi: 10.1677/joe.1.06880. PMID: 17210750

[167] Aditya S Rattan A . Sclerostin inhibition: a novel target for the treatment of postmenopausal osteoporosis. J Midlife Health. (2021) 12:267–75. doi: 10.4103/jmh.JMH_106_20. PMID: 35264832 PMC8849148

[168] Funck-Brentano T Cohen-Solal M . Anti-sclerostin antibodies in osteoporosis and other bone diseases. J Clin Med. (2020) 9. doi: 10.3390/jcm9113439. PMID: 33114755 PMC7694131

[169] Marom R Rabenhorst BM Morello R . Osteogenesis imperfecta: an update on clinical features and therapies. Eur J Endocrinol. (2020) 183:R95–R106. doi: 10.1530/EJE-20-0299. PMID: 32621590 PMC7694877

[170] Foulquier S Daskalopoulos EP Lluri G Hermans KCM Deb A Blankesteijn WM . WNT signaling in cardiac and vascular disease. Pharmacol Rev. (2018) 70:68–141. doi: 10.1124/pr.117.013896. PMID: 29247129 PMC6040091

[171] Bovijn J Krebs K Chen C-Y Boxall R Censin JC Ferreira T . Evaluating the cardiovascular safety of sclerostin inhibition using evidence from meta-analysis of clinical trials and human genetics. Sci Transl Med. (2020) 12. doi: 10.1126/scitranslmed.aay6570. PMID: 32581134 PMC7116615

[172] Khosla S Hofbauer LC . Osteoporosis treatment: recent developments and ongoing challenges. Lancet Diabetes Endocrinol. (2017) 5:898–907. doi: 10.1016/S2213-8587(17)30188-2. PMID: 28689769 PMC5798872

[173] Bone HG Dempster DW Eisman JA Greenspan SL McClung MR Nakamura T . Odanacatib for the treatment of postmenopausal osteoporosis: development history and design and participant characteristics of LOFT, the long-term odanacatib fracture trial. Osteoporos Int A J Establ As Result Coop Between Eur Found Osteoporos Natl Osteoporos Found USA. (2015) 26:699–712. doi: 10.1007/s00198-014-2944-6. PMID: 25432773 PMC4312384

[174] Dai R Wu Z Chu HY Lu J Lyu A Liu J . Cathepsin K: the action in and beyond bone. Front Cell Dev Biol. (2020) 8:433. doi: 10.3389/fcell.2020.00433. PMID: 32582709 PMC7287012

[175] Chen R Chen C Geng B Yang C Xiao H Yang F . Efficacy and safety of odanacatib for osteoporosis treatment: a systematic review and meta-analysis. Arch Osteoporos. (2023) 18:67. doi: 10.1007/s11657-023-01261-7. PMID: 37169994

[176] Lutgens E Lutgens SPM Faber BCG Heeneman S Gijbels MMJ de Winther MPJ . Disruption of the cathepsin K gene reduces atherosclerosis progression and induces plaque fibrosis but accelerates macrophage foam cell formation. Circulation. (2006) 113:98–107. doi: 10.1161/CIRCULATIONAHA.105.561449. PMID: 16365196

[177] Furuya Y Inagaki A Khan M Mori K Penninger JM Nakamura M . Stimulation of bone formation in cortical bone of mice treated with a receptor activator of nuclear factor-κB ligand (RANKL)-binding peptide that possesses osteoclastogenesis inhibitory activity. J Biol Chem. (2013) 288:5562–71. doi: 10.1074/jbc.M112.426080. PMID: 23319583 PMC3581422

[178] Törring O . Effects of denosumab on bone density, mass and strength in women with postmenopausal osteoporosis. Ther Adv Musculoskelet Dis. (2015) 7:88–102. doi: 10.1177/1759720X15579189. PMID: 26029270 PMC4426099

[179] Portal-Núñez S Mediero A Esbrit P Sánchez-Pernaute O Largo R Herrero-Beaumont G . Unexpected bone formation produced by RANKL blockade. Trends Endocrinol Metab. (2017) 28:695–704. doi: 10.1016/j.tem.2017.06.003. PMID: 28733136

[180] He Y Li Z Ding X Xu B Wang J Li Y . Nanoporous titanium implant surface promotes osteogenesis by suppressing osteoclastogenesis via integrin β1/FAKpY397/MAPK pathway. Bioact Mater. (2022) 8:109–23. doi: 10.1016/j.bioactmat.2021.06.033. PMID: 34541390 PMC8424426

[181] Karsdal MA Martin TJ Bollerslev J Christiansen C Henriksen K . Are nonresorbing osteoclasts sources of bone anabolic activity? J Bone Miner Res Off J Am Soc Bone Miner Res. (2007) 22:487–94. doi: 10.1359/jbmr.070109. PMID: 17227224

[182] Segovia-Silvestre T Neutzsky-Wulff AV Sorensen MG Christiansen C Bollerslev J Karsdal MA . Advances in osteoclast biology resulting from the study of osteopetrotic mutations. Hum Genet. (2009) 124:561–77. doi: 10.1007/s00439-008-0583-8. PMID: 18987890

[183] Silva BC Costa AG Cusano NE Kousteni S Bilezikian JP . Catabolic and anabolic actions of parathyroid hormone on the skeleton. J Endocrinol Invest. (2011) 34:801–10. doi: 10.3275/7925. PMID: 21946081 PMC4315330

